# LFA-1 activation enriches tumor-specific T cells in a cold tumor model and synergizes with CTLA-4 blockade

**DOI:** 10.1172/JCI154152

**Published:** 2022-07-01

**Authors:** Amber Hickman, Joost Koetsier, Trevin Kurtanich, Michael C. Nielsen, Glenn Winn, Yunfei Wang, Salah-Eddine Bentebibel, Leilei Shi, Simone Punt, Leila Williams, Cara Haymaker, Charles B. Chesson, Faisal Fa’ak, Ana L. Dominguez, Richard Jones, Isere Kuiatse, Amy R. Caivano, Sayadeth Khounlo, Navin D. Warier, Upendra Marathi, Robert V. Market, Ronald J. Biediger, John W. Craft, Patrick Hwu, Michael A. Davies, Darren G. Woodside, Peter Vanderslice, Adi Diab, Willem W. Overwijk, Yared Hailemichael

**Affiliations:** 1 Department of Melanoma Medical Oncology,; 2 Department of Translational Molecular Pathology, and; 3 Department of Lymphoma Myeloma, The University of Texas MD Anderson Cancer Center, Houston, Texas, USA.; 4 Molecular Cardiology Research Laboratories, Texas Heart Institute, Houston, Texas, USA.; 5 7 Hills Pharma, Houston, Texas, USA.; 6 Department of Biology and Chemistry, University of Houston, Houston, Texas, USA.

**Keywords:** Therapeutics, Cancer immunotherapy

## Abstract

The inability of CD8^+^ effector T cells (Teffs) to reach tumor cells is an important aspect of tumor resistance to cancer immunotherapy. The recruitment of these cells to the tumor microenvironment (TME) is regulated by integrins, a family of adhesion molecules that are expressed on T cells. Here, we show that 7HP349, a small-molecule activator of lymphocyte function–associated antigen-1 (LFA-1) and very late activation antigen-4 (VLA-4) integrin cell-adhesion receptors, facilitated the preferential localization of tumor-specific T cells to the tumor and improved antitumor response. 7HP349 monotherapy had modest effects on anti–programmed death 1–resistant (anti–PD-1–resistant) tumors, whereas combinatorial treatment with anti–cytotoxic T lymphocyte–associated protein 4 (anti–CTLA-4) increased CD8^+^ Teff intratumoral sequestration and synergized in cooperation with neutrophils in inducing cancer regression. 7HP349 intratumoral CD8^+^ Teff enrichment activity depended on CXCL12. We analyzed gene expression profiles using RNA from baseline and on treatment tumor samples of 14 melanoma patients. We identified baseline CXCL12 gene expression as possibly improving the likelihood or response to anti–CTLA-4 therapies. Our results provide a proof-of-principle demonstration that LFA-1 activation could convert a T cell–exclusionary TME to a T cell–enriched TME through mechanisms involving cooperation with innate immune cells.

## Graphical abstract

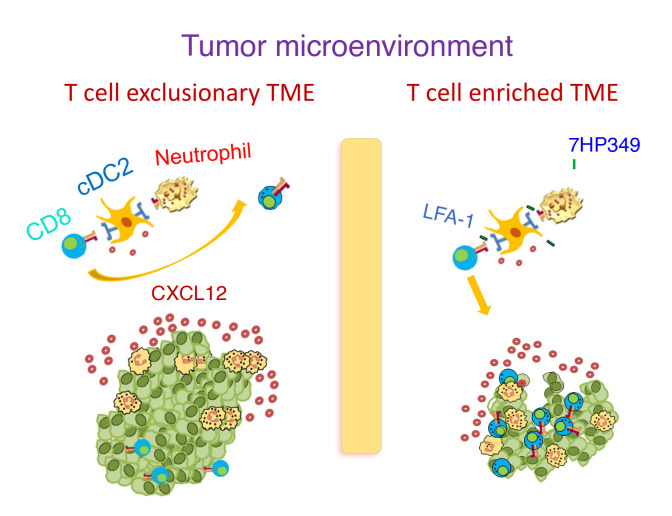

## Introduction

Immune checkpoint blockade (ICB) Abs that target programmed death 1 (PD-1) and cytotoxic T lymphocyte–associated protein 4 (CTLA-4), which are focused on activating and mobilizing the body’s immune system to eradicate malignant cells, have provided therapeutic benefits to patients with multiple malignancies, including melanoma, but a significant proportion of patients fail to benefit from such immunotherapies (1). Although multiple mechanisms of resistance to ICB have been implicated, the failure of effector T cells (Teffs) to migrate from the circulation to the tumor microenvironment (TME) has been identified repeatedly (2–5). Anti–PD-1/L1 therapies are less efficacious in noninflamed “cold” tumors, which are characterized by poor lymphocyte infiltration, rare programmed death ligand 1 (PD-L1) expression, increased immunosuppressive components, and abnormal angiogenesis in the TME (3, 4, 6).

Lymphocyte function–associated antigen-1 (LFA-1) (also known as α_L_β_2_) is critical to the recruitment of T cells to the TME. LFA-1 is constitutively expressed on leukocytes and normally exists in circulation in a low-affinity, inactive conformational state. Signaling via chemokine or immune receptors shifts LFA-1 into a high-affinity, active integrin conformation (7–9). Binding of ICAM-1 to LFA-1 facilitates endothelium adhesion, prolonged contact with antigen-presenting cells, and targeted cell killing (10, 11). Immunosuppressive factors, such as VEGF, downregulate the expression of ICAM-1 on the tumor endothelium, contributing to the lack of adhesiveness/localization of T cells (6, 12). Therefore, immune surveillance may fail as a result of defective cell adhesion (10, 13). Activation of LFA-1 on tumor-trafficking T cells to increase binding to ICAM-1 is a potential strategy to increase the recruitment of tumor-specific T cells to the TME (10, 11). Productive interactions between LFA-1 and ICAM-1 can result in cytotoxic T lymphocytes (CTLs) exiting the systemic circulation, infiltrating the tumor tissue, and triggering effector functions, resulting in tumor destruction.

THI0019 is a small molecule activator of very late activation antigen-4 (VLA-4) (also known as α_4_β_1_) and LFA-1 (14). THI0019 directly facilitates VLA-4–dependent lymphocyte rolling, firm adhesion, and migration on the VLA-4 ligands VCAM-1 and CS-1 (14). THI0019 also enhances LFA-1–dependent firm adhesion to ICAM-1 (14). From a mechanistic standpoint, a small molecule activator of VLA-4 and LFA-1 could have synergistic effects with ICB because of its potential to influence the immune response at multiple levels, including antigen presentation, immune cell trafficking to the TME, and Teff cytolytic activity. We previously demonstrated that the LFA-1/ICAM-1 axis is critical for anti–CTLA-4–activated CD8^+^ Teff localization and antitumor activity (15). Here, we show that the integrin-activating compound 7HP349, a structural analogue of THI0019 currently in clinical development (ClinicalTrials.gov NCT04508179), increases tumor-specific T cell activation and localization to a non–T cell–inflamed cold TME typically characterized by low ICAM-1 expression and enhances the antitumor activity of CTLA-4 and PD-1/L1 ICB therapies.

## Results

### 7HP349 increases antitumor response to CTLA-4 blockade.

**Figure 1:**
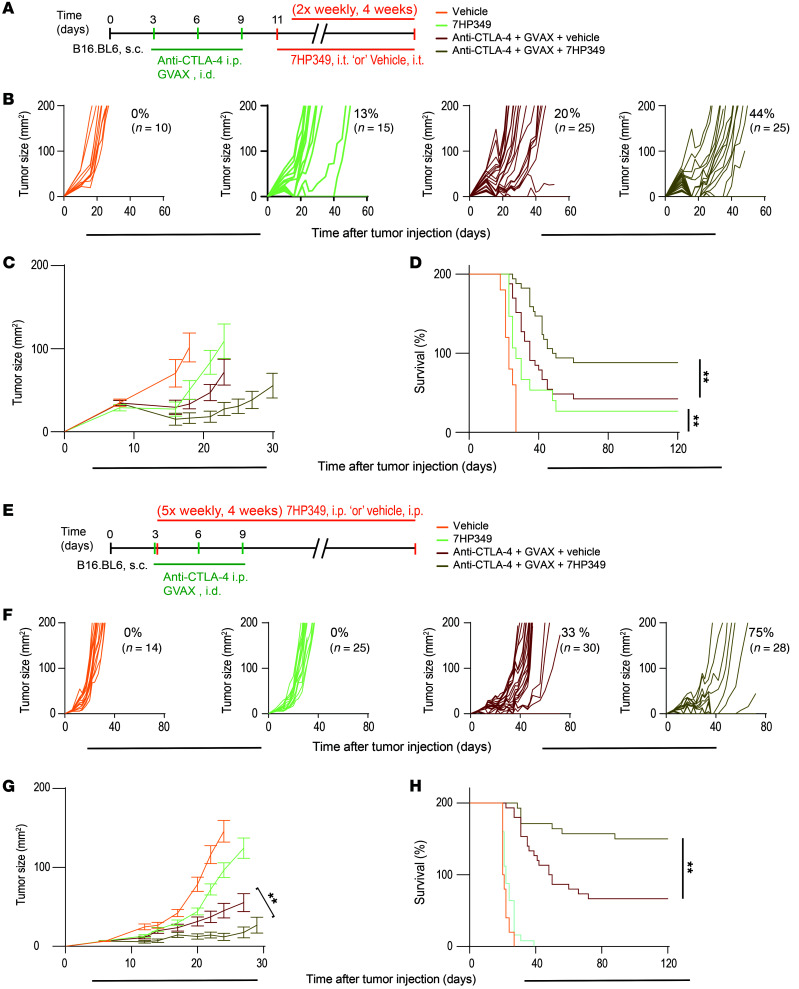
7HP349 increases antitumor response to CTLA-4 blockade. (See Supplemental Figures 1–3). (**A**–**D**) Female C57BL/6 mice received i.d. GVAX with i.p. anti–CTLA-4 3 days after B16.BL6 injection or i.t. vehicle or i.t. 7HP349 4 weeks (2 × weekly) after tumor injection, as indicated. (**A**) Treatment schematic. (**B**) Tumor growth curves of biologically independent mice by treatment group. (**C**) Average tumor burden (mean ± SEM). (**D**) Overall survival of the indicated treatment groups. **P < 0.05, log-rank test. (**E**–**H**) Mice were treated as in **A** but with systemic i.p. administration of 7HP349 or vehicle for 4 weeks (5 × weekly), as indicated. (**E**) Treatment schematic. (**F**) Tumor growth curves of biologically independent mice by treatment group. (**G**) Average tumor burden (mean ± SEM). **P < 0.01, nonparametric ANOVA, Kruskal-Wallis test. (**H**) Kaplan-Meier survival curve. **P < 0.01, log-rank test. Data are pooled from 2 independent experiments.

We used a previously described model of checkpoint blockade therapy for aggressive B16.BL6 melanoma (16), which formed the basis for the clinical trials that led to the FDA approval of anti–CTLA-4 therapy (17). This model includes anti–CTLA-4 and GM-CSF–producing B16.BL6 (GVAX) to amplify the weak endogenous immune response to B16.BL6. GVAX alone does not affect the growth of B16.BL6; therapeutic efficacy requires CTLA-4 blockade (17). To determine whether 7HP349 monotherapy improves the antitumor response to poorly immunogenic B16.BL6, we randomly assigned C57BL/6 mice bearing 10-day-old B16.BL6 to intratumoral (i.t.) 7HP349 or vehicle treatment. Once animals had developed tumors of approximately 25 mm^2^, they were injected with 7HP349 i.t. combined with anti–CTLA-4 i.p. and GVAX intradermal (i.d.). 7HP349 dosing resulted in a significant improvement in survival compared with vehicle alone (*P* < 0.05; Figure 1, A–D, with 2 of 15 (13%) mice treated with 7HP349 being tumor free compared with 0% of mice treated with vehicle control. Likewise, i.t. 7HP349, in combination with anti–CTLA-4, significantly improved median and tumor-free survival compared with anti–CTLA-4 plus vehicle (*P* < 0.05; Figure 1D). We performed 7HP349 i.p. dosing and observed no difference in therapeutic benefit between 7HP349 and vehicle (*P* > 0.05; Figure 1, E–H). Interestingly, anti–CTLA-4 antitumor efficacy was significantly enhanced in combination with 7HP349 compared with vehicle (77% versus 27%, *P* < 0.01; Figure 1H). We assessed the therapeutic effect of 7HP349 using a Lewis lung carcinoma (LLC1) model, another syngeneic non–T cell–inflamed tumor in the context of dual blockade of CTLA-4 and anti–PD-1. In this setting, vehicle and 7HP349 monotherapies dosed 2 times weekly for 2 weeks or dual ICB blockade and vehicle were not effective (0% of animals cured), while a combination of dual ICB blockade and i.t. 7HP349 cured 25% of the animals (*P* < 0.01; Supplemental Figure 1, A–D; supplemental material available online with this article; https://doi.org/10.1172/JCI154152DS1). In parallel with i.t. dosing, i.p. 7HP349 and dual ICB blockade cured 10% of the animals (*P* < 0.05, Supplemental Figure 1, E–H).

We determined the effects of 7HP349, anti–PD-1, and the combination of both in 3 syngeneic mouse models: CT26 (colon carcinoma) in BALB/c and E.G7-OVA (thymoma) and B16.BL6 in C57BL/6. BALB/c mice were injected with CT26 tumor cells on day 0, then were treated starting on day 8 with 5 doses of 7HP349 (or vehicle control), with or without 4 doses of anti–PD-1 (Supplemental Figure 2A). 7HP349 increased median survival both alone (20 versus 15 days, *P* < 0.0001) and in combination with anti–PD-1 (18 versus 15 days, *P* = 0.0407) compared with vehicle (Supplemental Figure 2, B–D). We found similar effects in anti–PD-1–resistant E.G7-OVA (Supplemental Figure 2, E–H) and B16.BL6 (Supplemental Figure 2, I–L) tumor models. To evaluate systemic immunity involving tumor growth at multiple tumor sites, we established a pulmonary metastasis model by i.v. injecting CT26 tumor cells that had been genetically modified to express firefly luciferase, which allows quantitation of live CT26 cells (tumor burden) by bioluminescence imaging (BLI). BLI analysis showed that 7HP349 delayed tumor progression, both as monotherapy and in combination with anti–PD-1 (Supplemental Figure 3, A and B). Together, these results indicate that 7HP349 has a therapeutic benefit in combination with standard CTLA-4, PD-1, or dual CTLA-4/PD-1 blockade for the treatment of cancer in murine tumor models.

### 7HP349 enhances T cell adhesion, cytolytic activity, cell spreading, migration, and costimulation.

**Figure 2:**
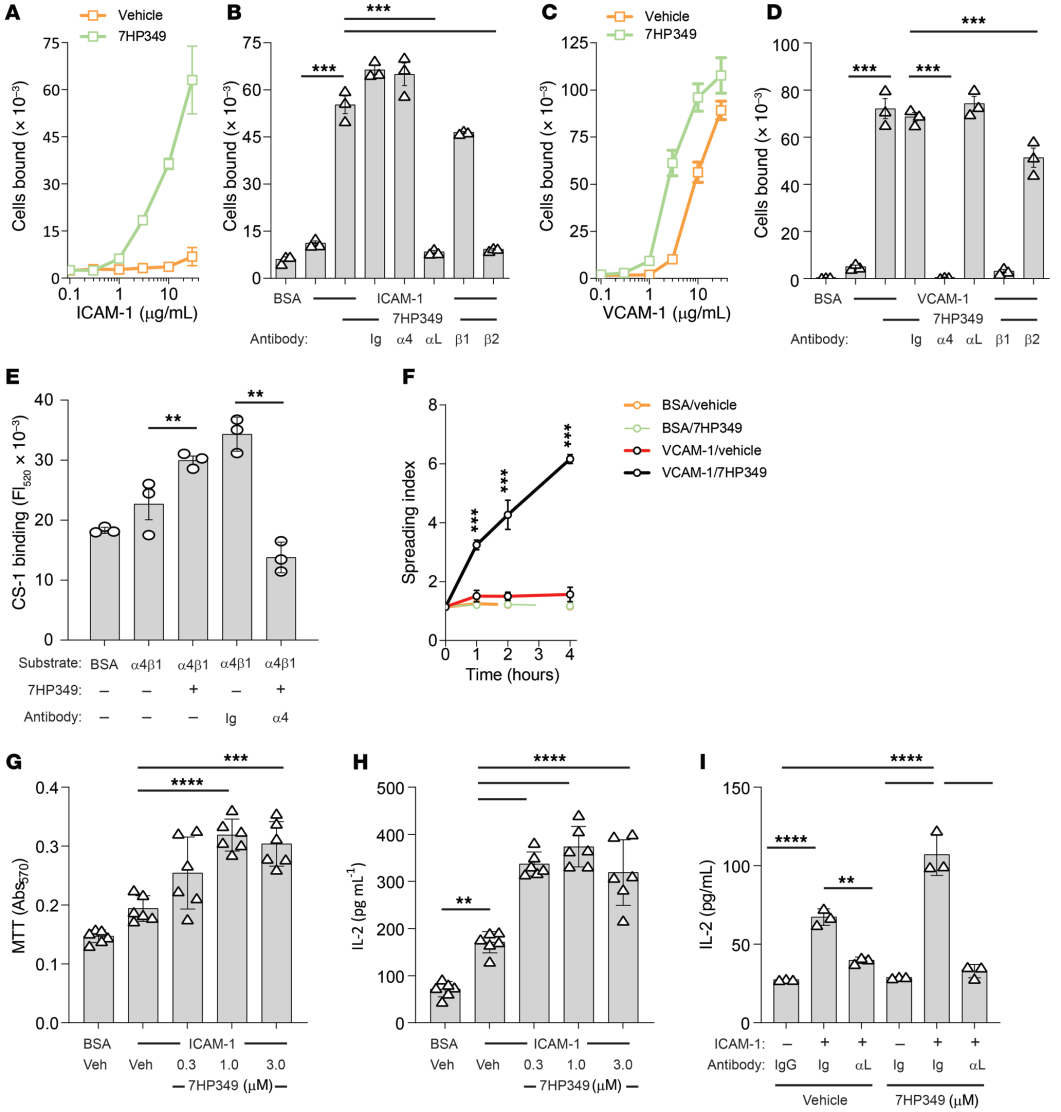
7HP349 enhances T cell adhesion, cell spreading, and costimulation. (See Supplemental Figures 4 and 5). (**A**–**D**) Purified T cell adhesion to indicated concentrations of plastic immobilized ligands VCAM-1 or ICAM-1. Data are represented as mean ± SD. ***P ≤ 0.001, Tukey’s test. (**E**) 7HP349 (30 μM) induced purified integrin α_4_β_1_ binding to ligand CS-1 (*n* = 3). (**F**) 7HP349 (10 μM) induced HSB cell spreading on α_4_β_1_ ligand VCAM-1. (*n* = 3) (**G**) Proliferation assays were performed with purified human T cells with mAb OKT3 and ICAM-1 immobilized at 5 ng/well and 200 ng/well, respectively (*n* = 6). Veh, vehicle. (**H**) IL-2 measurements were made by Elisa from supernatants collected from proliferation assays (*n* = 6). (**I**) T cell proliferation in the presence of 10 μg/mL function blocking mAb (*n* = 3) and control IgG. Data are represented as mean ± SD. **P ≤ 0.01; ***P ≤ 0.001; ****P ≤ 0.0001, Tukey’s test.

A key step in the conversion of the small molecule antagonist TBC3486 into the integrin activator THI0019 was the replacement of the carboxylic acid group with a methyl ester (Supplemental Figure 4A and ref. 14). Extensive structure-activity relationship data indicated that the bis(aryl)methylcarbamate group was sensitive to modification, although other aromatic groups could be substituted for the thiophene (Supplemental Figure 4A, compounds 1–3), consistent with molecular docking studies (Supplemental Figure 4B). In an effort to maximize the potential to activate integrin-mediated cell adhesion, a next-generation compound, 7HP349, was synthesized that displayed 2 bis(arylmethyl)carbamate groups in symmetrical fashion (Supplemental Figure 4A). In adhesion assays using purified peripheral blood human T cells, 7HP349 significantly and specifically enhanced integrin α_L_β_2_-mediated binding to ICAM-1 (Figure 2, A and B) and α_4_β_1_ binding to VCAM-1 (Figure 2, C and D). 7HP349 directly enhanced purified integrin α_4_β_1_ binding to its ligand CS-1 (Figure 2E). A similar integrin selectivity to THI0019 was also observed (Supplemental Figure 4, C–E, and ref. 14). Off-target effects, such as nonspecific interaction with chemokine receptors, which could indirectly activate integrins, was ruled out, as 7HP349 enhanced integrin-dependent cell adhesion in the presence of pertussis toxin (Supplemental Figure 4, F and G). 7HP349 did not induce the binding of ligand-induced binding site (LIBS) Abs, which bind the β subunit, supporting a mode of binding in which 7HP349 does not engage the metal ion-dependent adhesion site and does not act like a ligand mimetic (Supplemental Figure 4H). Facilitating development of 7HP349 is the observation that it is equally potent across multiple species (Supplemental Figure 4I).

We evaluated whether 7HP349 can enhance CTL cytotoxic capacity against HLA-matched autologous melanoma tumor cells by measuring active caspase-3 in tumor target cells (18). 7HP349 increased T cell tumor target killing in a dose-dependent manner (Supplemental Figure 5A). We assessed ICAM-1 (CD54) and VCAM-1 (CD106) expression on melanoma cells by flow cytometry because they may influence responsiveness to 7HP349 therapy. We observed distinct ICAM-1 expression, while VCAM-1 was undetectable (Supplemental Figure 5B), consistent with the results of our previous report showing that ICAM-1 can be constitutively expressed whereas VCAM-1 expression is induced by therapy in an IFN-γ–dependent manner (15). To further demonstrate the impact of 7HP349 on ligand engagement, the compound was assayed for its effect on cell spreading. The human T cell line HSB was incubated with either vehicle or 7HP349 in 96-well plates coated with BSA or VCAM-1. 7HP349 did not induce spreading of cells on BSA (Figure 2F). However, the rate and extent of spreading on VCAM-1 was significantly higher in the presence of 7HP349 than vehicle (Figure 2F). In standard Transwell chemotaxis assays, 7HP349 enhanced CXCL12-dependent Jurkat cell chemotaxis on α_4_β_1_ and α_L_β_2_ ligands VCAM-1 and ICAM-1, respectively (Supplemental Figure 5, C and D). To determine the effects of 7HP349 on integrin costimulation in vitro, purified peripheral blood human T cells were plated on ICAM-1 and the anti-TCR/CD3 complex mAb OKT3. 7HP349 enhanced integrin α_L_β_2_-dependent T cell proliferation and costimulation of IL-2 production (Figure 2, G–I). In summary, 7HP349 enhances T cell adhesion mediated by both VLA-4 and LFA-1 integrins and augments the functional consequences of integrin engagement, such as increased cell migration, costimulation of T cell activation, and Teff cytolytic activity.

### 7HP349 treatment increases myeloid and lymphoid cell localization to tumors.

**Figure 3:**
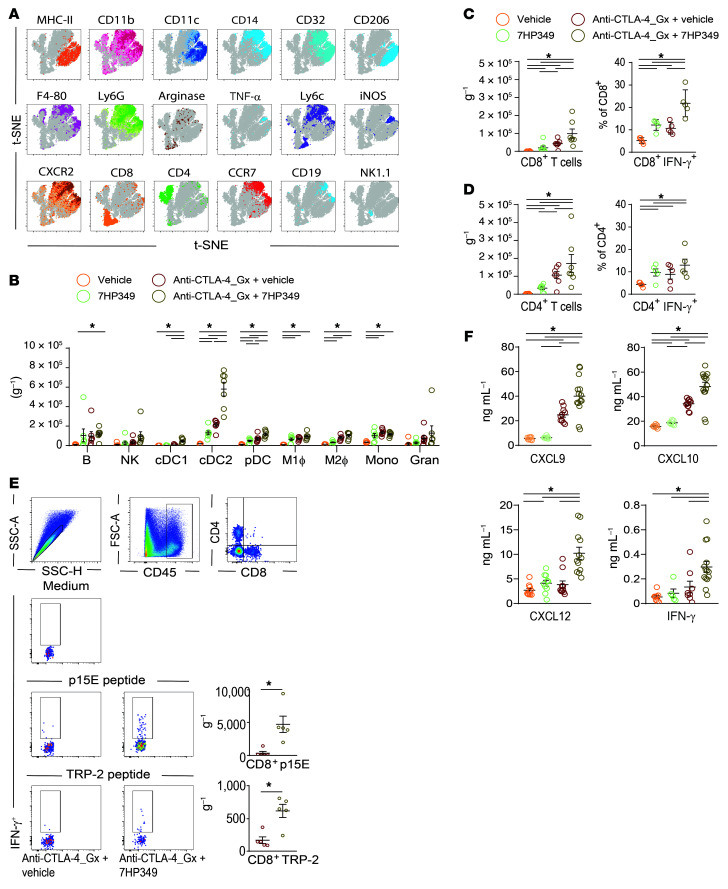
7HP349 treatment affects tumor myeloid and lymphoid cell composition. (See Supplemental Figures 6-9). Mice bearing 3-day s.c. B16.BL6 tumor received 7HP349 i.p. or vehicle or GVAX i.d. with anti–CTLA-4 i.p., as described in Figure 1E. Tumors were harvested 21 days after injection. (**A**) t-SNE plots of CD45^+^ B16.BL6 tumor-infiltrating myeloid cells with expression of selected markers. (**B**) Frequencies of myeloid cell subsets, adjusted per tissue weight (g^–1^) across treatment groups (*n* = 5). (**C**) Frequency of CD8^+^ Teffs (CD44^hi^CD11a^hi^), adjusted per tissue weight (g^–1^) (*n* = 7), or percentage of IFN-γ^+^ polyclonal CD8^+^ Teffs (*n* = 5). (**D**) Frequency of CD4^+^ Teffs (CD44^hi^CD11a^hi^), adjusted per tissue weight (mg^–1^) (*n* = 7), or percentage of IFN-γ^+^ polyclonal CD4^+^ Teffs in tumor (*n* = 5). (**E**) CD8^+^IFN-γ^+^ p15E- or TRP-2–specific Teffs (mean ± SEM), adjusted per tissue weight (g^–1^) (*n* = 5, *P < 0.05, unpaired *t* test). (**F**) Cytokine and chemokine concentrations in supernatant from tumors (*n* = 10–12). Data in **B**–**D** and **F** are represented as mean ± SEM. Analyses were performed using 1-way ANOVA, Tukey’s test. *P < 0.05.

We assessed the impact of 7HP349 on modulating tumor myeloid cell composition. The TME contains heterogeneous populations of myeloid cells that can exert immunosuppressive or immunostimulatory effects (19–22). To comprehensively profile tumor immune-cell infiltrates, we derived a flow cytometry panel that encompasses immune cell heterogeneity, mirroring a representative panel of multiparameter CyTOF (Figure 3A). In a combinatorial therapeutic regimen comprising 7HP349, vehicle, or anti–CTLA-4, CD11b^hi^CD11C^+^CD8^–^( cDC2), CD11b^lo^CD11c^+^B220^+^ (pDC), CD11b^+^F4-80^+^TNF-α^+^iNOS^+^ (M1 macrophages), CD11b^+^F4-80^+^CD206^+^ (M2 macrophages), and CD11b^+^Ly6c^hi^ (inflammatory monocytes [IMs]) frequencies were significantly higher in the TME in mice treated with 7HP349 than in those treated with vehicle (Figure 3B). Anti–CTLA-4 in combination with 7HP349 increased DC subsets, such as pDC, cDC1, and cDC2 (Figure 3B).

We next quantified the frequency of tumor-infiltrating T cells in mice bearing the poorly immunogenic B16.BL6 tumor. We found a significant increase in the frequency of IFN-γ^+^CD44^hi^CD11a^hi^ CD8^+^ Teffs (*P* < 0.05; Figure 3C) and CD4^+^ Teffs (*P* < 0.05; Figure 3D) in tumors from mice treated with 7HP349 compared with vehicle or in combination with anti–CTLA-4. The CD4^+^ and CD8^+^ Teff frequency detected within the TME was significantly higher than that detected in other organs (Supplemental Figure 6). To further define the specificity of tumor-infiltrating CD8^+^ T cell clones, we gated on CD8^+^ T cells that recognize TRP-2 and p15E melanoma epitopes. Remarkably, we detected higher frequencies of TRP-2 and p15E-epitope–specific CD8^+^ T cells in tumors from mice that received CTLA-4 blockade in combination with 7HP349 than in those that received CTLA-4 blockade and vehicle (Figure 3E). To understand which pathways drive immune suppression and limit T cell activity beyond CTLA-4, we examined expression of the inhibitory markers PD-1, Tim-3, Lag-3, KLRG-1, and CTLA-4 in spleen- (Supplemental Figure 7A) and tumor-infiltrating CD8^+^ and CD4^+^ (Supplemental Figure 7B) Teffs at day 21. We found that 7HP349 treatment elicited significantly reduced CTLA-4 expression on both CD4^+^ and CD8^+^ T cells compared with vehicle, with no statistically detectable differences in other inhibitory receptors between treatment groups (Supplemental Figure 7, A and B).

Immune cell recruitment to the TME correlated with the expression of myeloid cell attractant chemokines, such as C-C motif ligand 12 (CCL12), CCL17, CCL19, CCL20, CCL21; C-X-3-C ligand 1 (CX3CL1); the T cell–attractant chemokines C-X-C motif ligand 10 (CXCL10), CXCL11, and CXCL12; and Th1 cytokines IFN-γ, IL-12p70, and IL-2 (Figure 3F and Supplemental Figures 8 and 9).

**Figure 4:**
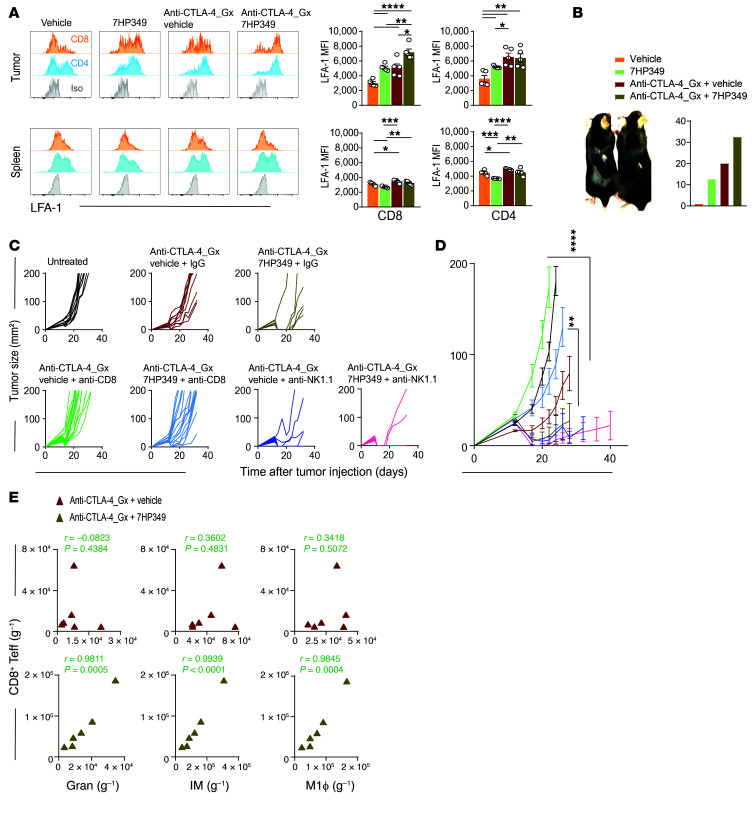
7HP349 enhances CD8^+^ Teff preferential localization to tumor. (See Supplemental Figures 10–12). (**A**) Mice were treated as in Figure 3A. LFA-1 expression on CD8^+^ or CD4^+^ Teffs from tumor and spleen tissues (*n* = 5). Data are represented as mean ± SEM, 1-way ANOVA, Tukey’s test. *P < 0.05; **P < 0.01; ***P < 0.001; ****P < 0.0001. (**B**) Vitiligo expression in mice (left) and the percentage of mice with vitiligo (right). (**C** and **D**) C57BL/6 mice bearing 3-day-old s.c. B16.BL6 tumors received i.p. 7HP349 or vehicle or i.d. GVAX with i.p. anti–CTLA-4, as described in Figure 1E, and mAb depletion of CD8^+^ T cells or NK cells on days 3, 5, 7, 9, and 11 after tumor injection (*n* = 10). (**C**) Tumor growth curves of biologically independent mice by treatment group. (**D**) Average tumor burden (mean ± SEM) for mice treated with anti–CTLA-4_GX plus vehicle (IgG versus anti-CD8, ****P < 0.0001) or anti–CTLA-4_GX plus 7HP349 (IgG versus anti-CD8, **P < 0.01), 1-way ANOVA, Tukey’s test. (**E**) Correlation analysis of granulocytes, IMs, or M1 macrophages versus CD8^+^ Teffs from tumors of mice treated with anti–CTLA-4 and 7HP349 or vehicle (*n* = 6).

To assess whether 7HP349 treatment modulates CD4^+^ and CD8^+^ Teff integrin surface expression in a tissue-specific manner, we compared LFA-1 MFI on T cells isolated from tumors, vaccine-draining lymph node (VdLN), PBMCs, or spleens (Supplemental Figure 10). LFA-1 expression was higher on tumor-localizing Teffs than in the aforementioned tissues (Supplemental Figure 10). Notably, 7HP349 alone induced increased LFA-1 expression in tumor-infiltrating CD8^+^ Teffs to levels seen with anti–CTLA-4 alone. Also, 7HP349 in combination with anti–CTLA-4 further augmented LFA-1 expression higher on CD8^+^ Teffs localizing to tumor than to the aforementioned tissues (Figure 4A and Supplemental Figure 10).

As evidence of enhanced immune cell–mediated antitumor activity, we observed an increased incidence of vitiligo expression at the tumor injection site in mice treated with standard checkpoint blockade therapy in combination with 7HP349 compared with vehicle (Figure 4B). To identify the target Teffs, we examined the role of CD8^+^ T cells and NK cells in B16.BL6-bearing C57BL/6 mice. Antitumor effects in B16.BL6 mice were abrogated only when CD8^+^ T cells were depleted, without an apparent increase in tumor burden associated with NK cell depletion (Figure 4, C and D). Although CD8^+^ T cell cytotoxicity is critical for antitumor response, mice treated with 7HP349 showed less CD8^+^ Teff dependency than did the vehicle group (Figure 4, C and D), indicating that 7HP349 can generate other antitumor compensatory mechanisms driven by other immune cells.

To determine whether myeloid cell composition at the TME associated with the antitumor response, we correlated tumor immune cell infiltrate frequencies or ratios with tumor burden. Tumor burden was estimated by tumor weight at day 21 of tissue harvest. We generated 8 variables that could be tested for their association with tumor burden (Supplemental Figure 11A). We found that cDC2/Treg, CD8^+^ Teff/Treg, and CD4^+^ Teff/Treg ratios were significantly increased in tumors from mice treated with 7HP349 compared with vehicle, and these ratios were negatively correlated with tumor burden in mice treated with 7HP349 (Supplemental Figure 11B). These differences were not detected in mice treated with vehicle. Interestingly, we found a significant negative correlation between tumor weight and CD8^+^ Teffs, CD4^+^ Teffs, cDC2, or neutrophil frequency in mice treated with CTLA-4 blockade in combination with 7HP349 (Supplemental Figure 11A). Furthermore, the correlation of granulocytes, IMs, or M1 macrophages and CD8^+^ Teffs (Figure 4E) or CD4^+^ Teffs (Supplemental Figure 12) showed a high concordance and was statistically significant when these were measured in the setting of 7HP349 treatment (Figure 4E and Supplemental Figure 12). Given the immunosuppressive potential of granulocytic cells (23), the finding that granulocytes are negatively correlated with tumor burden and show high concordance with TIL frequencies in the setting of 7HP349 treatment is intriguing. Together, these results indicate that 7HP349 may induce tumor inhibition through both innate and adaptive immune cells.

### VCAM-1 blockade induces enhancement of DCs homing to the TME.

**Figure 5:**
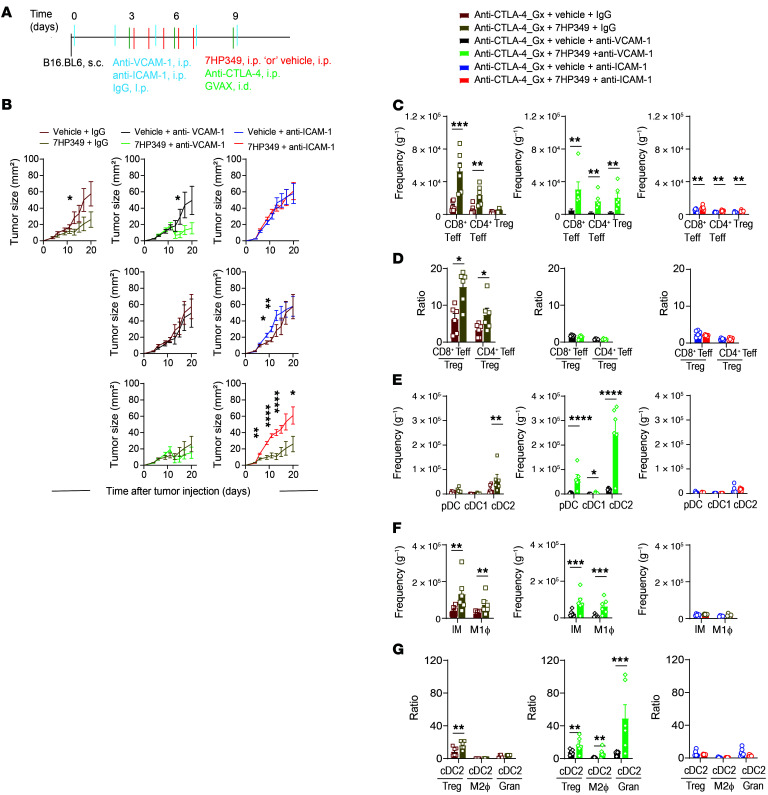
VCAM-1 blockade induces enhancement of DC homing to the TME. (See Supplemental Table 1). Mice bearing 3-day s.c. B16.BL6 received anti–CTLA-4 therapy and 7HP349 or vehicle and/or anti-ICAM-1, anti-VCAM-1, or IgG, as indicated. (**A**) Experimental schematic. (**B**) Average tumor burden after IgG, anti–VCAM-1, or anti–ICAM-1 treatment (*n* = 10). Data are represented as mean ± SEM, 1-way ANOVA, Tukey’s test. *P < 0.05; **P < 0.01; ****P < 0.0001. (**C**) Frequency of CD8^+^ or CD4^+^ Teffs and Tregs adjusted per tissue weight (mg^–1^) in mice after IgG, anti–VCAM-1, or anti–ICAM-1 treatment (*n* = 6). (**D**) CD4^+^ Teff/Treg and CD8^+^ Teff/Treg ratios following IgG, anti-VCAM-1, or anti–ICAM-1 treatment (*n* = 6). (**E**) Frequency of IMs, M1 macrophages (M1Ф), pDC, cDC1, and cDC2 adjusted per tissue weight (mg^–1^) in mice after IgG, anti–VCAM-1, or ICAM-1 treatment (*n* = 6). (**F**) cDC2/Treg, cDC2/M2 macrophage, cDC2/granulocyte, and cDC2/monocyte ratios after IgG, anti–VCAM-1, or anti–ICAM-1 treatment (*n* = 6). (**G**) M1 macrophages/M2 macrophages (M2Ф) and IM/M2 macrophage ratios after IgG, anti–VCAM-1, or anti–ICAM-1 treatment (*n* = 6). Data shown in **C**–**G** are represented as mean ± SEM. Analyses were performed using unpaired *t* test. *P < 0.05; **P < 0.01; ***P < 0.001; ****P < 0.0001.

To evaluate the relative importance of LFA-1 or VLA-4 on immune Teff retention at the TME, we quantitated the frequency of myeloid and T cells by flow cytometry following Ab blockade of ICAM-1 or VCAM-1 together with CTLA-4 blockade (Figure 5A). Because previous mouse mechanistic studies reported that CD8^+^ Teff entry into the TME is dependent on LFA-1/ICAM-1 adhesion (15), we were particularly interested in whether VLA-4/VCAM-1 adhesion can further augment immune cell retention. Ab blockade of VCAM-1 did not affect antitumor response in vehicle-treated mice compared with 7HP349-treated mice, indicating that tumors grew similarly in mice following VCAM-1 blockade or in mice in the control group treated with 7HP349 compared with vehicle (Figure 5B). Consistent with our previous finding (15), ICAM-1 blockade resulted in abrogation of the antitumor response, with no noteworthy difference between mice treated with 7HP349 or vehicle (Figure 5B). The frequency of CD4^+^ Teffs and CD8^+^ Teffs at the TME was reduced by 2-fold after VCAM-1 blockade and by 90% after ICAM-1 blockade compared with control (Figure 5C). 7HP349 treatment increased CD4^+^ Teff/Treg or CD8^+^ Teff/Treg ratios in control mice, while these ratios did not change with ICAM-1 or VCAM-1 blockade (Figure 5D).

Next, we determined whether ICAM-1 or VCAM-1 blockade induces changes in myeloid cell composition. Interestingly, we found that 7HP349 treatment resulted in a greater than 23-fold increase in pDC after VCAM-1 blockade compared with ICAM-1 blockade or greater than 5-fold compared with control, a greater than 5-fold increase in CD8^+^ DCs (cDC1) after VCAM-1 blockade compared with ICAM-1 blockade treated, or no detectable change compared with control mice (Figure 5E). Similarly, cDC2 increased more than 17-fold after VCAM-1 blockade compared with ICAM-1 blockade or more than 7-fold compared with control (Figure 5E and Supplemental Table 1). In contrast, vehicle treatment did not change pDC, cDC1, or cDC2 frequency after VCAM-1 blockade or ICAM-1 blockade or in control mice, indicating that DC retention at the TME is induced by 7HP349, which is positively correlated with antitumor response (Figure 5B). 7HP349 enhanced IM and M1 macrophage numbers, and ICAM-1 blockade partially blocked this (Figure 5F and Supplemental Table 1). Furthermore, we observed an increase in the cDC2/Treg ratio in mice receiving anti–CTLA-4 with 7HP349 compared with vehicle (Figure 5G) that was not inhibited with VCAM-1 blockade. Taken together, these results indicate that, while the LFA-1/ICAM-1 axis is critical for T cell and DC homing, the VLA-4/VCAM-1 axis could be a roadblock for DCs homing to the TME. However, its blockade did not hinder the therapeutic efficacy of 7HP349.

### Neutrophils are critical for CD8^+^ Teff i.t. sequestration and antitumor response in 7HP349-treated mice.

**Figure 6:**
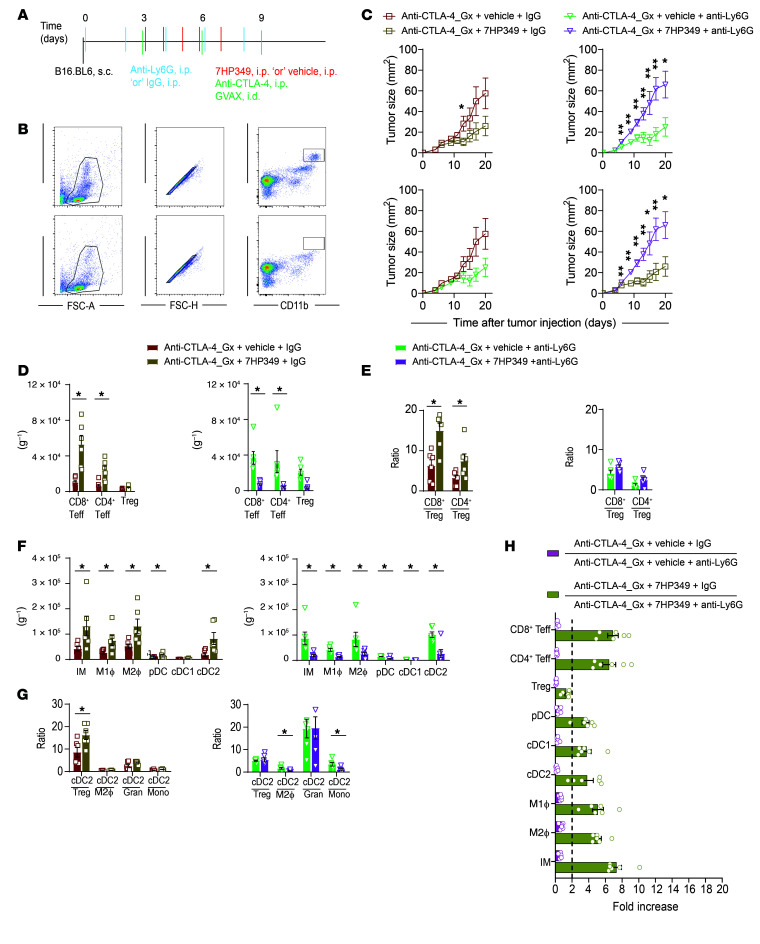
Neutrophils are critical for CD8^+^ Teff antitumor response in 7HP349-treated mice. (See Supplemental Figure 13). Mice bearing 3-day s.c. B16-BL6 melanomas received anti–CTLA-4 therapy and 7HP349 or vehicle and/or anti-Ly6G mAb or IgG, as indicated. (**A**) Experimental schematic. (**B**) Flow cytometry analysis showing anti-Ly6G mAb depletion of neutrophils at day 5 in PBMCs. FSC-A, forward scatter–A; FSC-H, forward scatter–H. (**C**) Average tumor burden in mice (*n* = 10) after IgG or anti-Ly6G treatment. Data are represented as mean ± SEM.One-way ANOVA, Tukey’s test. *P < 0.05; **P < 0.01. (**D**) Frequency of CD8^+^ or CD4^+^ Teffs and Tregs, adjusted per tissue weight (mg^–1^) in mice after IgG or anti-Ly6G treatment (*n* = 6). (**E**) CD8^+^ Teff/Treg and CD4^+^ Teff/Treg ratios following IgG or anti-Ly6G treatment (*n* = 6). (**F**) Frequency of IMs, M1 macrophages, pDC, cDC1, and cDC2 adjusted per tissue weight (mg^–1^) in mice after IgG or anti-CXCL12 treatment (*n* = 6). (**G**) cDC2/Tregs, cDC2/M2 macrophage, cDC2/granulocyte, or cDC2/monocyte ratios after IgG or anti-Ly6G treatment (*n* = 6). (**H**) Immune cell sequestration fold increase at the TME after IgG or anti-Ly6G treatment (*n* = 5). Data are represented as mean ± SEM. Data analyses (**D**–**G**) were performed using unpaired *t* test. *P < 0.05.

The observed strong correlation between neutrophils and CD8^+^ Teffs and its corresponding negative association with tumor burden in mice undergoing 7HP349 treatment (Figure 4E and Supplemental Figure 11A) prompted us to determine the extent to which the perceived cooperation between CD8^+^ Teffs and neutrophils is critical for the CD8^+^ T cell–dependent antitumor response (Figure 4D). To determine the role of neutrophils on 7HP349-mediated antitumor response, we used selective neutrophil depletion with mAb 1A8 (anti-Ly6G) (Figure 6, A and B). Depletion of neutrophils in vehicle-treated mice did not affect B16.BL6 tumor growth (Figure 6C). In contrast, depletion of neutrophils in 7HP349-treated mice resulted in a significantly reduced effect of 7HP349 therapy — the tumors grew more rapidly when neutrophils were depleted. Consistent with the B16.BL6 tumor–bearing mouse findings, neutrophil depletion in LLC1 tumor–bearing mice treated with dual CTLA-4 and PD-1 blockade resulted in LLC1 tumors growing more slowly in mice receiving vehicle treatment and more rapidly in mice receiving 7HP349 (Supplemental Figure 13). These results indicate that neutrophil responsiveness to LFA-1 activation via 7HP349 treatment could become a viable strategy for reshaping their function and potential antitumor response.

Next, we assessed the impact of neutrophil depletion on the selective retention of CD8^+^, CD4^+^, and cDC2 over that of Tregs in the TME. In the absence of neutrophils, 7HP349 treatment did not result in an increase in frequency of CD8^+^ and CD4^+^ T cells (Figure 6D), pDC, cDC1, cDC2, M1 macrophages, M2 macrophages, and monocytes (Figure 6F) and did not increase the ratios of CD8^+^ Teffs/Tregs, CD4^+^ Teffs/Tregs, and cDC2/Tregs in neutrophil-depleted mice compared with neutrophil-competent (control) mice (Figure 6, E and G), indicating that 7HP349’s effect on promoting the accumulation of CD4^+^CD8^+^ Teffs and antigen-presenting cells within the TME is dependent on neutrophils. This observation is in line with previous research by others showing that activated neutrophils produce important proinflammatory cytokines and chemokines that attract mononuclear cells (24). In fact, the expression of key neutrophil-secreted chemokines, including CCL3, CCL4, CCL20, and CXCL12, was increased in the tumor supernatants of mice treated with 7HP349 (Figure 3F and Supplemental Figure 9). In addition, our analysis of tumor cell infiltrates revealed neutrophils’ role in facilitating complex remodeling of the lymphoid and myeloid cell landscape, including more than 6-fold in CD8^+^ and CD4^+^ Teffs and more than 3-fold in cDC2 i.t. sequestration in 7HP349-treated mice (Figure 6H).

**Figure 7:**
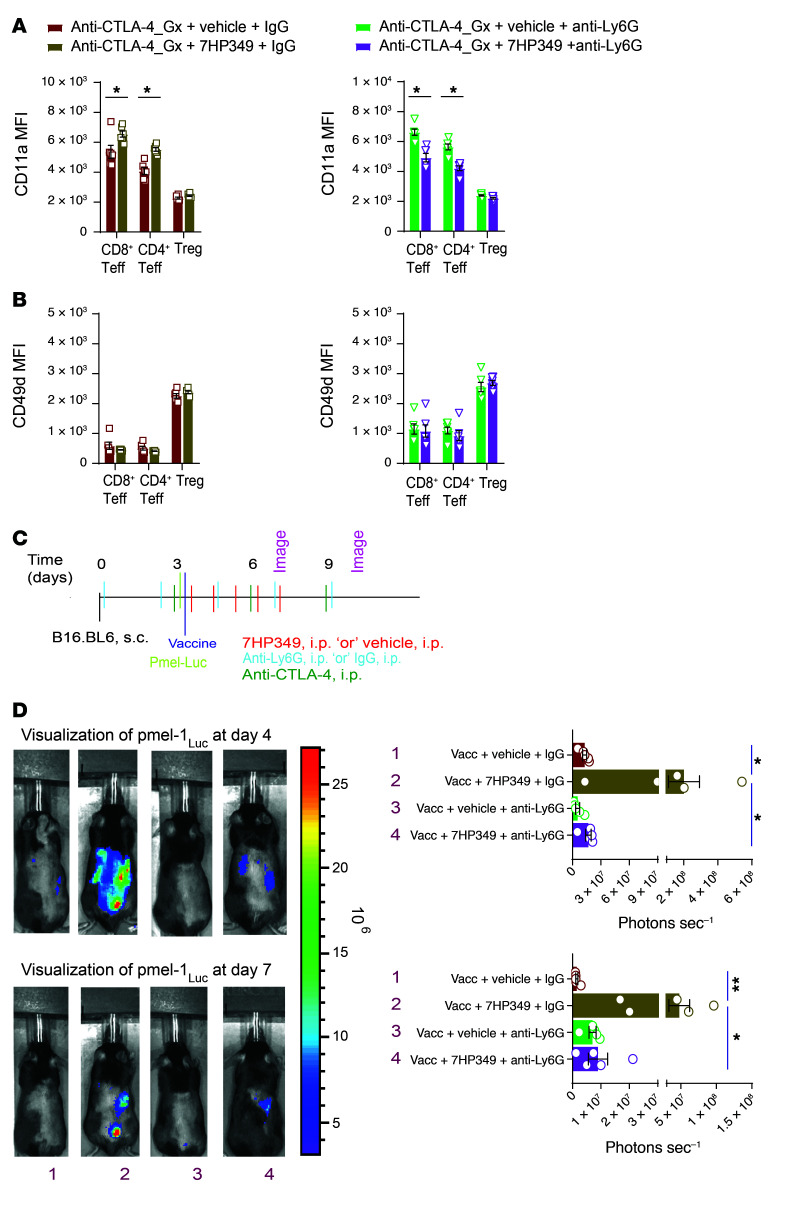
CD8^+^ Teff i.t. sequestration is dependent on neutrophils in 7HP349-treated mice. (**A**) Mice treated as in Figure 6A. CD11a expression on CD8^+^ and CD4^+^ Teff after IgG or anti-Ly6G treatment (*n* = 6). (**B**) CD49d expression on CD8^+^ and CD4^+^ Teffs after IgG or anti-Ly6G treatment (*n* = 6). Data in **A** and **B** are represented as mean ± SEM. *P < 0.05, unpaired *t* test. (**C** and **D**) Mice bearing 4-day s.c. B16 tumors received 6-day–cultured V-effLuc–transduced pmel-1 T cells i.v. after vaccination with gp100/saline and anti–CTLA-4 i.p. and/or 7HP349, vehicle, IgG, or anti-Ly6G, as indicated. (**C**) Treatment schematic. (**D**) V-effLuc–transduced pmel-1 T cells are visualized by whole mouse imaging 4 days (top panel) and 7 days (bottom panel) after vaccination. Combination of bar and dot plots showing absolute pmel-1 T cell luminescence (photons s^–1^). Data are represented as mean ± SEM. *n* = 5. Analyses were performed using 1-way ANOVA, Tukey’s test. *P < 0.05; **P < 0.01.

Given that 7HP349 acts through activation of LFA-1 and VLA-4, we determined the extent to which 7HP349 directly affects CD11a or CD49d expression on granulocytic immune cells. We found no change in the expression of these molecules on neutrophils, regardless of treatment with 7HP349 or vehicle (data not shown). Conversely, CD11a expression on CD8^+^ Teffs and CD4^+^ Teffs decreased (Figure 7A) in neutrophil-deficient mice, while CD49d expression remained unchanged (Figure 7B), indicating that the effect of 7HP349 on expression of CD11a on CD8^+^ and CD4^+^ Teffs is finely regulated by the presence of neutrophils.

Next, to assess the impact of neutrophils on CD8^+^ Teff i.t. sequestration, we examined homing patterns after the adoptive transfer of luciferase-expressing pmel-1 CD8^+^ T cells (with TCR specificity to gp100 melanoma epitope). We previously used this technique to track pmel-1 T cell homing to tumor and inflamed vaccination sites (25). Luciferase-expressing pmel-1 Teffs, cultured for 6 days, were transferred i.v. into C57BL/6 mice 6 days after tumor injection with concurrent gp100 peptide in saline. In addition, mice received the covax costimulatory combination of anti-CD40, high-dose IL-2, and a TLR-7 agonist (imiquimod) cream. This approach allowed us to track antigen-specific T cell localization to the tumor, spleen, liver, or cutaneous vaccine injection sites. Consistent with the flow cytometry results, we observed overwhelming pmel-1 CD8^+^ Teff sequestration at the tumor and cutaneous vaccine injection sites in neutrophil-competent mice, but none in neutrophil-depleted mice (Figure 7, C and D). Taken together, these observations indicate that 7HP349 treatment reshapes neutrophils’ role as critical for regulating CD8^+^ Teff tumor homing and antitumor activity.

### CXCL12 is required for LFA-1 activation and CD8^+^ Teff i.t. sequestration.

**Figure 8:**
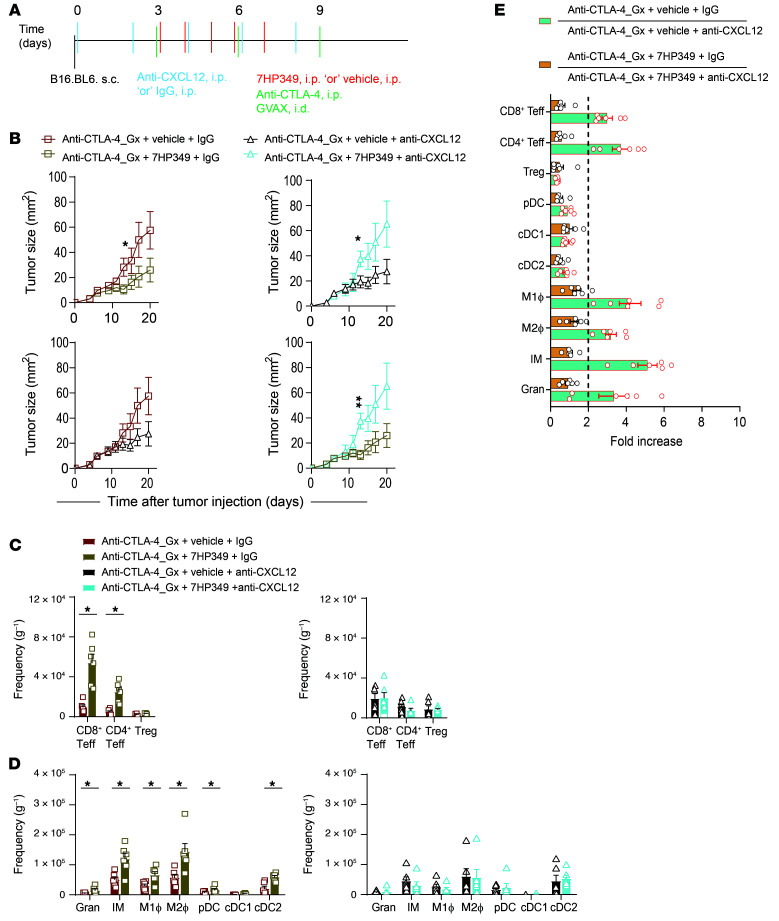
CXCL12 is required for CD8^+^ Teff i.t. sequestration. (See Supplemental Figures 14 and 15). Mice bearing 3-day s.c. B16-BL6 melanomas received anti–CTLA-4 therapy and 7HP349 or vehicle and/or anti-CXCL12 or IgG, as indicated. (**A**) Treatment schematic. (**B**) Average tumor burden in mice (*n* = 10) after IgG or anti-CXCL12 treatment. Data are represented as mean ± SEM. One-way ANOVA, Tukey’s test. *P < 0.05; **P < 0.01. (**C**) Frequency of CD8^+^ Teffs, CD4^+^ Teffs, and Tregs adjusted per tissue weight (mg^–1^) in mice after IgG or anti-CXCL12 treatment (*n* = 6). (**D**) Frequency of IMs, M1 macrophages, M2 macrophages, pDC, cDC1, and cDC2 adjusted per tissue weight (mg^–1^) in mice after IgG or anti-CXCL12 treatment (*n* = 6). (**E**) Immune cell sequestration fold increase at the TME after IgG or anti-CXCL12 treatment (*n* = 6). Data in **C** and **D** are represented as mean ± SEM. Analyses were performed using unpaired *t* test. *P < 0.05.

Under inflammatory conditions, neutrophils release a variety of cytokines, one of which is CXCL12, which was significantly elevated in tumor supernatant from B16.BL6-bearing mice that had been treated with anti–CTLA-4 and 7HP349 (Figure 3F), for which chemokine receptor CXCR4 is expressed on CD8^+^ Teffs (Supplemental Figure 8). Although multiple myeloid cells show CXCL12 expression in the TME (Supplemental Figure 14, A and B), neutrophils are a significant source of CXCL12 (Supplemental Figure 14, C–E). Next, to identify the molecular mechanism that mediates the cooperation between neutrophils and CD8^+^ Teffs, we focused on neutrophil-secreted CXCL12. A previous study elegantly demonstrated that CD8^+^ T cells trafficking to inflamed airways followed the CXCL12 chemokine trail left by neutrophils (26). In addition, studies by our group and others confirmed that the CXCR3/CXCL9 axis is critical for CD8^+^ Teff trafficking to the TME and antitumor response (10); likewise, we determined whether CXCR4/CXCL12-mediated chemotaxis has a role in CD8^+^ Teff accumulation and antitumor response. C57BL/6 mice bearing B16.BL6 tumors received CTLA-4 blockade and 7HP349, together with CXCL12-neutralizing mAb or IgG (Figure 8A). We found less tumor control in mice treated with anti-CXCL12 than with IgG (control) in the setting of 7HP349, but not vehicle (Figure 8B). Similarly, CXCL12 neutralization experiments performed in the LLC1 model showed anti–CTLA-4 antitumor response is dependent on CXCL12 in the setting of 7HP349 (Supplemental Figure 15). CXCL12 neutralization abolished the 7HP349 treatment–related increase in frequencies of CD8^+^ and CD4^+^ Teffs (Figure 8C) and tumor myeloid cell infiltrates consisting of IMs, granulocytes, M1 macrophages, M2 macrophages, pDC, and cDC2 (Figure 8D). In contrast, we detected no apparent CXCL12 effect on cDC1 recruitment to the TME, regardless of whether mice were treated with 7HP349 or vehicle (Figure 8D). Since we did not observe CXCR4 expression on macrophages and IMs (data not shown), the reduced recruitment of these immune cells in the setting of CXCL12 depletion is intriguing and may be secondary to factors produced by the recruited CXCR4^+^ cells. Together, our results show that 7HP349 treatment increased i.t. sequestration of T cells and myeloid cells in a CXCL12-dependent manner (Figure 8E).

**Figure 9:**
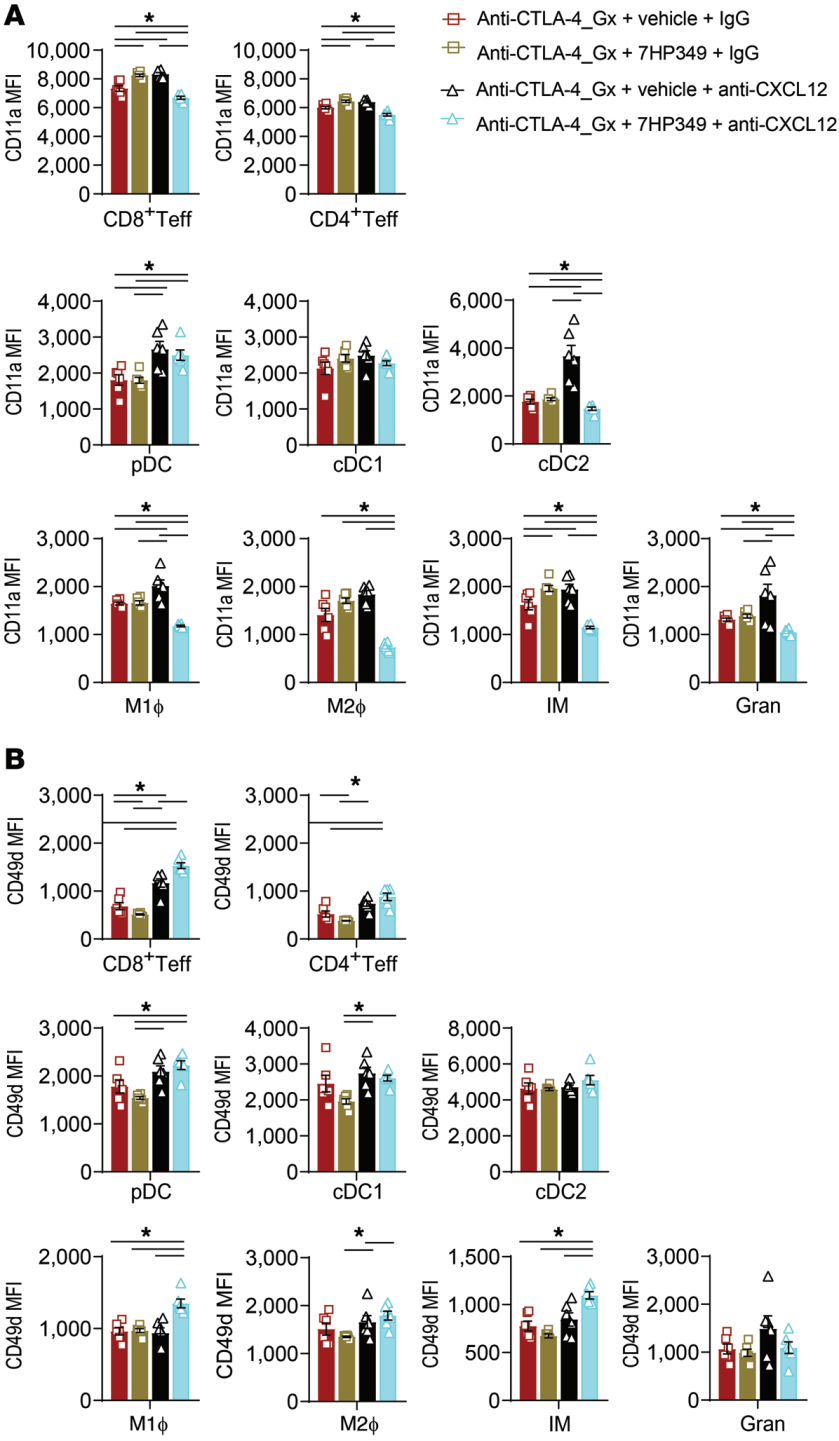
CXCL12 is required for LFA-1 activation at the TME in 7HP349-treated mice. Mice were treated as in Figure 8A. (**A**) CD11a (α_L_) integrin expression (*n* = 5) on leukocytes at the TME, as determined by a flow cytometry analysis following IgG or anti-CXCL12 treatment (*n* = 6). (**B**) CD49d (α_4_) integrin expression on leukocytes at the TME, as determined by a flow cytometry analysis following IgG or anti-CXCL12 treatment (*n* = 6). Data are represented as mean ± SEM. Analyses were performed using 1-way ANOVA, Tukey’s test. *P < 0.05.

To understand the mechanism whereby CXCL12 modulates T and myeloid cell sequestration, we examined CD11a and CD49d expression on T and myeloid cells from B16.BL6-bearing mice treated with anti–CTLA-4 and 7HP349, vehicle, anti-CXCL12, or IgG or combination therapy. Notably, expression of CD11a followed a similar trend and decreased on CD8^+^ Teffs, CD4^+^ Teffs, cDC2s, M1 macrophages, M2 macrophages, IMs, and granulocytes (Figure 9A) and remained unchanged on cDC1s and increased on pDC (Figure 9A) in mice after treatment with CXCL12-depleting Abs. In contrast, CD49d expression increased on CD8^+^ Teffs, CD4^+^ Teffs, pDC, cDC1s, M1 macrophages, and IMs (Figure 9B), but remained unchanged on cDC2s, M2 macrophages, and granulocytes (Figure 9B) in CXCL12-deficient mice undergoing 7HP349 treatment. Together, these results indicate that 7HP349’s effects involve CXCL12 in modulating CD8^+^ Teff homeostatic stability and crosstalk with monocytes and granulocytes via LFA-1/ICAM-1–mediated cell adhesion at the TME (26, 27).

### CXCL12 gene expression signature predicts response to CTLA-4 checkpoint blockade in melanoma.

**Figure 10:**
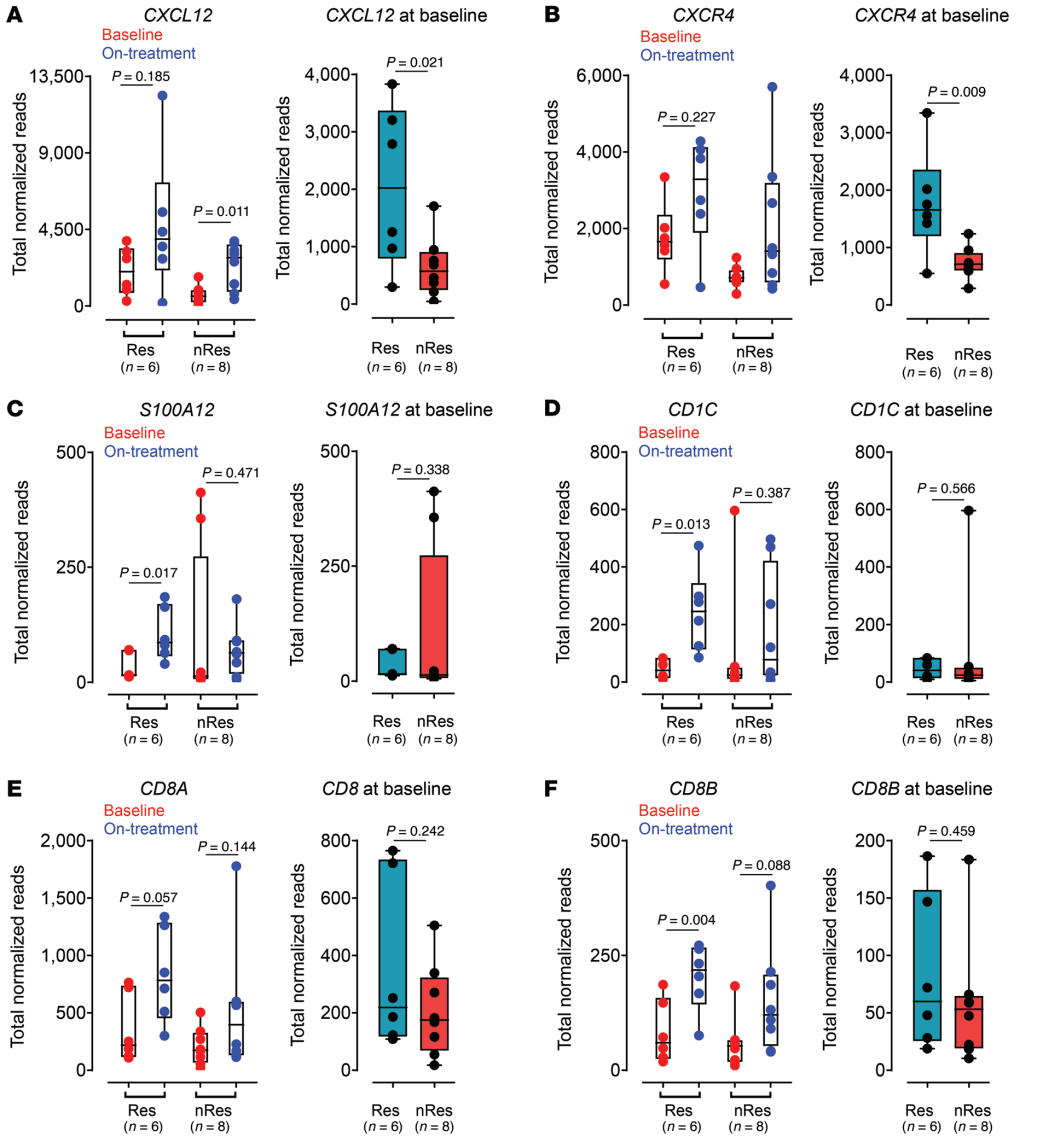
CXCL12 gene expression signatures predict response to CTLA-4 checkpoint blockade in melanoma. Human cancer patients (*n* = 14) are stratified based upon best overall response (BOR), as indicated in each graph. Individual gene expression changes between baseline and on-treatment 8 weeks after treatment with ipilimumab and tilsotolimod (TLR9 agonist). Box plots show individual gene normalized expression at baseline and week 8 in the local injected lesions. (**A**) *CXCL12*; (**B**) *CXCR4*; (**C**) *S100A12*; (**D**) *CD1C*; (**E**) *CD8A*; (**F**) *CD8B*. *P* values indicate significance using parametric *t* test (left panels) and nonparametric test (right panels). Data are presented as median, and whiskers on the box plots extend minimum to maximum points. The top and bottom lines of the box plots represent the interquartile range (IQR), the midline represents the median, and the whiskers on the box plots represent minimum and maximum values. Data analyses were performed for responders (Res) and nonresponders (nRes). Baseline versus on-treatment, paired *t* test; baseline alone, unpaired *t* test.

We next asked whether critical immune mechanisms underlying the core features of 7HP349’s effect on CTLA-4 checkpoint blockade antitumor response in our preclinical model were associated with a specific treatment outcome for anti–CTLA-4 therapy in patients with metastatic melanoma. We used the NanoString nCounter PanCancer Immune Profiling Panel (NanoPCIP) to understand immune-relevant gene expression in tumor tissue of patients with metastatic melanoma. We separated a cohort of patients into 2 groups: (a) patients with melanoma responding to anti–CTLA-4 (per RECISTv1.1) (*n* = 6); and (b) nonresponding to anti–CTLA-4 (*n* = 8). The mean total normalized reads for each gene at baseline (responding versus nonresponding tumor) were compared (Figure 7). Among the genes significantly upregulated in the responding tumors at baseline, interestingly, were genes that encode for *CXCL12* and *CXCR4* (Figure 10, A and B). Other genes markedly upregulated in expression following treatment were genes encoding for DCs *CD1C*, neutrophils *S100A12*, and CD8^+^ T cells *CD8B* (Figure 10, C–F). The Cancer Genome Atlas (TCGA) mRNA data analysis of tumor resections from patients with advanced melanoma (28) indicated higher *CXCR4* pretreatment expression in patients responding compared with those not responding to treatment. Similarly, *CXCR4*, *CD8A*, and *CD8B* pretreatment levels are associated with therapy benefits (Supplemental Figure 16). These data suggest that CD8^+^ T cells, neutrophils, cDC2s, and CXCL12 have more prevalent roles in modulating objective antitumor response.

### 7HP349 preserves immunologic memory upon tumor rechallenge.

**Figure 11:**
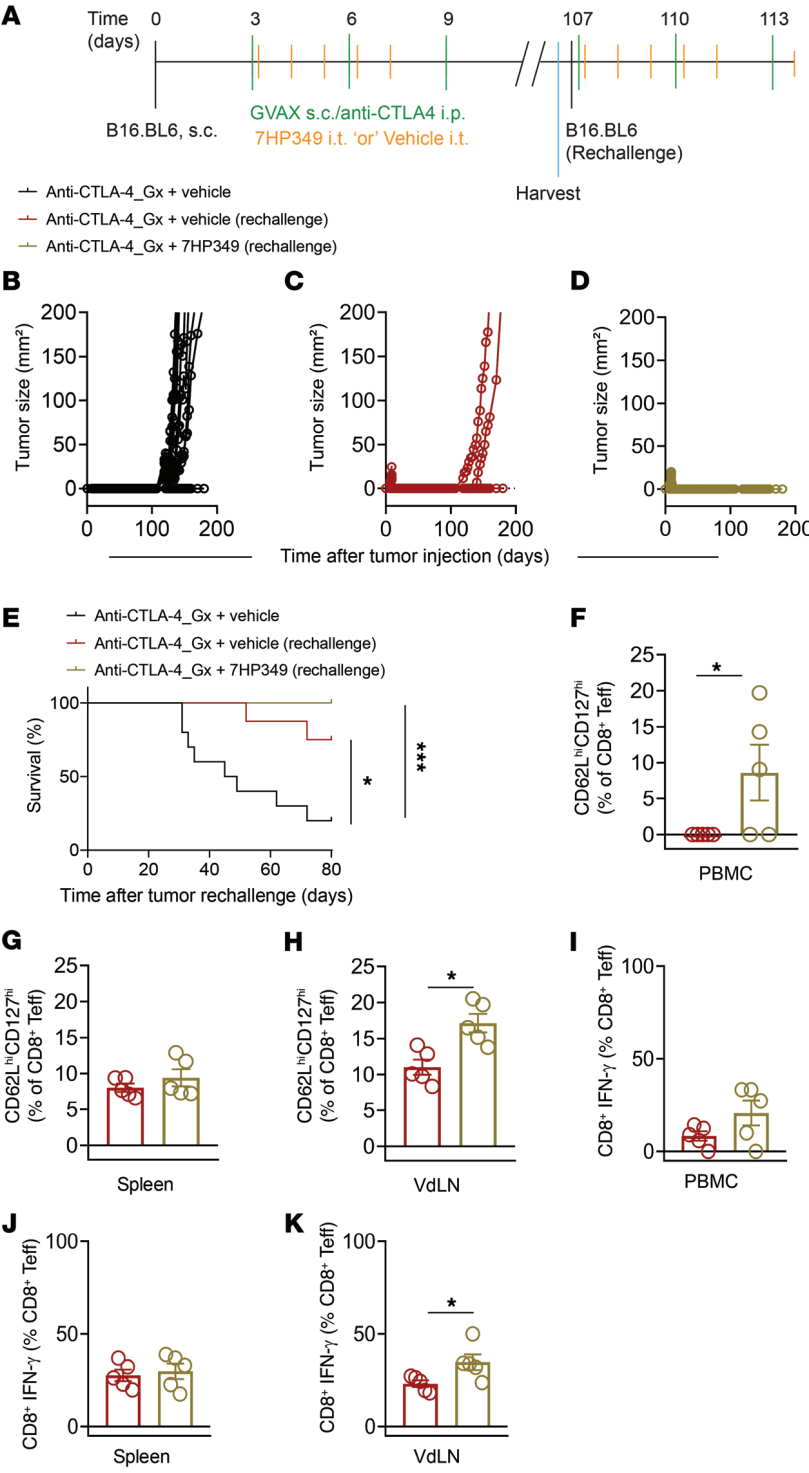
7HP349 preserves immunological memory upon tumor rechallenge. C57BL/6 mice, 3 days after s.c. injection with 3 × 10^4^ B16.BL6 cells, received 7HP349 or vehicle i.p. at days 3, 4, 5, 6, and 7 with GVAX i.d. and anti–CTLA-4 i.p. at days 3, 5, 7, and 9. PBMCs, VdLN, and spleen were harvested on day 100. (**A**) Experimental schematic shows the initial treatment schedule as well as the timing of the rechallenge. (**B**–**E**) The figure shows representative mice from each group. Age-matched naive, *n* =10; vehicle treated, *n* = 9; 7HP349 treated *n* = 10. (**B**) Tumor burden in age-matched treatment-naive control mice, (**C**) vehicle-treated mice, (**D**) and 7HP349-treated mice. (**E**) Kaplan-Meier survival curve. *P < 0.05; ***P < 0.001, log-rank test. (**F**–**H**) CD8^+^ central memory cells (TCM) in PBMCs, spleen, and VdLN (*n* = 5). (**I**–**K**) CD8^+^ IFN-γ^+^ T cells in PBMCs, spleen, and VdLN (*n* = 5). Data in **F**–**K** are represented as mean ± SEM. Analyses were performed using unpaired *t* test. *P < 0.05.

The generation of long-term T cell memory responses is important for an effective and durable antitumor response. Previous studies have shown that LFA-1/ICAM-1 interaction may be important in the generation of T cell immunologic memory (10). We evaluated the effect of 7HP349 in combination with anti–CTLA-4 on the formation of memory responses. Mice that had been confirmed to have been tumor free for 107 days after initial tumor challenge were compared against age-matched naive mice that received an anti–CTLA-4 boost 3 days after B16.BL6 injection (Figure 11A). The tumor rejection rates were 20% in the age-matched control, 60% in mice previously treated with vehicle plus anti–CTLA-4, and 100% in mice previously treated with 7HP349 plus anti–CTLA-4 (Figure 11, B–E). Immune profiling of CD8^+^ T cells in PBMCs, spleen, and VdLNs showed 7HP349 treatment–induced polarization of antigen-experienced T cells, predominantly toward the central memory phenotype (CD8^+^CD62L^hi^CD127^hi^) (Figure 11, F–H), with increased expression of IFN-γ in VdLNs and no detectable difference in PBMCs and spleen (Figure 11, I–K). Together, these results indicate that 7HP349 preserves immunologic memory response against B16.BL6.

## Discussion

While PD-1/L1 immune checkpoint blockade has been a major breakthrough in clinical cancer therapy, a significant drawback of this immunotherapeutic strategy remains the lack of efficient tumor localization of antitumor cells (3, 4). Tumor vessels are often poorly activated because of constitutive proangiogenic signaling in the TME and therefore constitute barriers to efficient leukocyte recruitment (13). As a result, mobilized cancer-fighting immune cells fail to migrate from the circulation to reach the site of tumor growth (5, 6). Herein, we demonstrate a therapeutic strategy using 7HP349 to increase ICB-induced CD8^+^ Teff i.t. sequestration and antitumor response. 7HP349-induced CD8^+^ Teff i.t. sequestration and antitumor activity were dependent on LFA-1, CXCL12, and neutrophils. In addition, 7HP349 promoted systemic immunologic memory and facilitated innate and adaptive immune cell cooperation within the TME.

For PD-1/L1 blockade therapy to induce tumor regression, preexisting antitumor CD8^+^ T cells that are negatively regulated by PD-1/ L1 –mediated adaptive immune resistance must be present (3, 29). In the current study, 7HP349 therapeutic effect in combination with anti–PD-1 was modest given the absence of a specific antigen target to stimulate CD8^+^ T cell antitumor response in host C57BL/6 and BALB/c mice. We show that 7HP349 in combination with anti–CTLA-4 and GVAX tumor vaccine results in synergistic, CD8^+^ T cell–dependent antitumor response in B16.BL6. Clinically, we anticipate that a combination of 7HP349 and PD-1/L1 blockade could potentially result in robust antitumor response, given that the host immune system in many melanoma patients is latently active and, when checkpoint brakes are removed, can durably clear neoplastic cells.

We identified several requirements for 7HP349 to mediate the regression of tumors that typically present with poor T cell infiltration. The immunomodulatory effect of 7HP349 was abrogated with the depletion of either CD8^+^ Teffs, Ly6G^+^ neutrophils, or CXCL12. Neutrophil sequestration at the TME via the LFA-1/ICAM-1–dependent pathway was intertwined with CD8^+^ Teffs; this was potentially mediated through CXCL12 crosstalk. Whole-body imaging provided evidence that Ly6G^+^ neutrophils influence CD8^+^ T cell accumulation at the TME. Of note, the accumulation of pmel-1 CD8^+^ T cells with TCR specificity to the gp100 melanoma epitope was reduced in mice treated with neutrophil depletion. These results align with those of recent reports that neutrophils recruit CD8^+^ T cells, as demonstrated in infections (26) and in cancer (30). A previous study reported that neutrophils and CD8^+^ T cell crosstalk are mediated by LFA-1/ICAM-1 interactions (30). In fact, the same study showed that the favorable significance of CD8^+^ T cell infiltration of colorectal cancer is significantly enhanced by concomitant infiltration by CD66b^+^ neutrophils (30). The effect of 7HP349 therapy on the increase in checkpoint blockade efficacy is also explained by the correlative immunologic data. Our analysis of TME immune infiltrates revealed that CD8^+^ Teff accumulation is associated with an increase in neutrophil, M1 macrophage, and IM infiltration to the TME. Furthermore, our results demonstrate neutrophils’ crucial role in promoting the recruitment of T cells, DCs, monocytes, and macrophages to the TME. The same findings indicate that Treg infiltration to the TME is reduced, resulting in increased CD4^+^ Teff, CD8^+^ Teff, and cDC2/Treg ratios (31).

Recent advances in T cell–based immunotherapy have revolutionized treatment strategies for multiple types of malignancies; however, approaches that rely on CD8^+^ Teffs alone could have limited success. Our results show that 7HP349 treatment–activated CD8^+^ Teffs generated outside the tumor stroma require Ly6G^+^ neutrophils for recruitment to the tumor site. Indeed, tumors containing neutrophils accumulated CD8^+^ Teffs efficiently. When examining the factors that could contribute to CD8^+^ Teff tumor recruitment, we found that CD8^+^ Teffs expressed the chemokine receptors CXCR3 and CXCR4, while the TME produced abundant CXCL9 and CXCL12, which are the key chemokines known to bind CXCR3 and CXCR4, respectively. The findings from our neutrophil depletion study in the LLC1 tumor model provided evidence that neutrophils are a major source of CXCL12 at the TME. Indeed, we confirmed that an increased i.t./stromal CXCL12 level was associated with therapeutic benefits in patients with melanoma undergoing anti–CTLA-4 immunotherapy (Figure 7A). Therefore, assessing the presence or absence of CXCL12 in tumors could be relevant for predicting patients’ clinical outcomes and defining treatment options. Our preclinical mouse model predicts that human cancer patients with low levels of CXCL12 and neutrophils could benefit from immunotherapies if they were treated concurrently with 7HP349. Conceivably, 7HP349 therapy, which increases CD8^+^ Teff recruitment to the TME in cooperation with neutrophils in a CXCL12-dependent manner, could trigger efficient tumor infiltration by CD8^+^ Teffs and increase the percentage of cancer patients who experience a response to immunotherapy. It should be noted that activation of LFA-1 via 7HP349 could also contribute to neutrophil trafficking to the tumor, leading to the subsequent recruitment of Teffs.

Our current study demonstrates that the 7HP349 effect promotes CXCL12-dependent immune cell sequestration at the TME, which could represent a new means of enhancing T cell homing that could be combined with immunotherapy strategies, such as checkpoint blockade and cancer vaccines in a T cell–noninflamed “cold” TME (4). Of note, 7HP349 antitumor therapeutic action required CXCL12. Conversely, CXCL12 depletion improved the ICB antitumor response in the absence of 7HP349 treatment. This is in line with the results of a previous study showing overexpression of CXCL12 in B16 melanoma–repelling antigen-specific T cells (32), suggesting complex and fine-tuned control of Teff infiltration by CXCL12/CXCR4 signaling. We previously reported that sequestration of specific T cells at inflamed vaccination sites resulted in dysfunctional T cells that were unable to traffic to tumors to cause tumor destruction (25). Here, we show that 7HP349 integrin activation reversed CXCL12’s effects on tumor-specific CD8^+^ Teffs, promoting CD8^+^ Teff persistence and functionality, as evidenced by an increase in IFN-γ secretion. CD8^+^ Teff IFN-γ secretion at the immunologic synapse could improve neutrophil survival, which is otherwise short (33); in addition, it promoted CXCL12-mediated feed-forward cooperative interaction with M1 macrophages and IMs at the TME (34). Although it has previously been reported that neutrophils can secrete and chemoattract CD8^+^ Teffs via CXCL12, the tumor stroma could not be ruled out as the main source; this question was not addressed in the current study and would be important to address as part of a future investigation. On the basis of these findings, we propose that the tumor stroma represents a myeloid cell–enriched microenvironment with abundant expression of CXCL12, which upon LFA-1 activation by 7HP349, could enhance recruitment and stabilize CD8^+^ Teffs at the immunologic synapse (9). This neutrophil-guided and LFA-1–mediated sequestration of CD8^+^ Teffs at the TME could further trigger cooperation with cDC2s, M1 macrophages, and IMs, which together become a potent antitumor arsenal.

## Methods

### Mice and tumor cells.

Female pmel-1 TCR transgenic mice on a C57BL/6 background (The Jackson Laboratory) were crossed with CD90.1 congenic mice to yield pmel-1^+/+^ × CD90.1^+/+^ mice. Female C57BL/6J and BALB/c mice were purchased from the Charles River Laboratory. Murine cell lines B16.BL6 and GM-CSF–producing B16.BL6 (GVAX) cell lines were gifts from P.M. Sharma (University of Texas MD Anderson Cancer Center). CT26 and E.G7-OVA cell lines were obtained from ATCC. The LLC1 cell line was a gift from M.A. Cortez (University of Texas MD Anderson Cancer Center). All cell lines were maintained in DMEM supplemented with 100 μg ml^–1^ penicillin, 100 μg ml^–1^ streptomycin, 50 mg ml^–1^ gentamycin, 2 mM l-glutamine, and 8% FCS (Invitrogen). EG7.OVA cells were maintained in the presence of G418 (EMD Millipore). The C57BL/6 strain was used for B16.BL6, EG7, and LLC1 and the BALB/c strain for CT26.

### Treatment.

The backs of mice were shaved and 100 μl of PBS s.c. containing 2.5 × 10^4^ B16.BL6 melanoma, 3 × 10^5^ E.G7-OVA, 1 × 10^6^ CT26, or 5 × 10^5^ LLC cells were injected. The B16.BL6-bearing mice were treated (s.c., posterior costal region) with 1 × 10^6^ irradiated (160 Gy) GVAX, together with 3 × 200 μg (10 mg kg^–1^), 100 μg (5 mg kg^–1^ body weight), and 100 μg (5 mg kg^–1^) anti–CTLA-4 (9H10, Bio X Cell) or anti–PD-L1 (10F.9G2, Bio X Cell) at the indicated time points. C57BL/6 mice bearing E.G7-OVA or LLC1 or BALB/c mice bearing CT26 tumors received similar treatment, with the omission of GVAX.

In the i.t. treatment approach, mice were administered 25 μl (1 mg kg^–1^) of i.t. 7HP349 or vehicle 2× biweekly, for a total of 4 weeks. In the i.p. treatment approach, mice received 50 μl (1 mg kg^–1^) of 7HP349 or vehicle on days 3, 4, 5, 6, and 7. Some mice were administered i.p. 200 μg (10 mg kg^–1^) anti-CD8 (clone 53-6.7, Bio X Cell), anti-NK1.1 (clone PK136), anti–ICAM-1 (clone YN1/1.7.4, Bio X Cell), anti–VCAM-1 (clone M/K-2.7, Bio X Cell), anti-Ly6G (clone 1A8, Bio X Cell), anti-CXCL12 (clone 79014, R&D Systems), or IgG at the indicated time points.

E.G7-OVA cells were injected s.c. into C57BL/6 mice. Mice received 200 μg anti–PD-1 i.p. on days 6, 8, 10, and 12 and 50 μl (1 mg kg^–1^) of 7HP349 or vehicle on days 6, 7, 8, 9, and 10. CT26 cells (1 × 10^6^) were injected s.c. into BALB/c mice on day 0. Mice received anti–PD-1 on days 8, 10, 12, and 14 and 50 μl (1 mg kg^–1^) of i.p. 7HP349 or vehicle on days 8, 9, 10, 11, and 12. The s.c. LLC1 tumor–bearing mice received 200 μg anti–CTLA-4 and 200 μg anti–PD-1 i.p. on days 5, 7, 9, and 11 and 25 μl (1 mg kg^–1^) of i.t. 7HP349 or vehicle 2× biweekly, for a total of 2 weeks, or 50 μl (1 mg kg^–1^) of 7HP349 or vehicle on days 5, 6, 7, 8, and 9.

### Peptide vaccination.

The synthetic, high-affinity H-2D^b^–restricted heteroclitic mouse gp100_25–33_ peptide (hgp100, KVPRNQDWL) was purchased from CPC Scientific at purity levels of more than 95%. Mice received 1000 naive pmel-1 T cells i.v. and were immunized with 2 separate s.c. injections at the base of the tail or in each flank with 100 μl of saline containing 100 μg hgp100_25–33_ peptide, and received covax (15), consisting of 100 μl 1× s.c. 500 μg/ml of CD40-specific mAbs (clone FGK4.5, Bio X Cell), 25 mg 1× topical imiquimod cream 5% (Aldara, Fougera), and a total of 100 μl 5× i.p. 1 × 10^6^ IU/ml rhIL-2 (TECIN, Hoffman La Roche Inc.) on days 0, 1, and 2.

### Murine cellular isolation, Ab staining, flow cytometry and CyTOF.

Mice were euthanized by CO_2_ inhalation. PBMCs were collected by tail bleed or cardiac puncture. Spleens, lymph nodes, and tumors were harvested and stored in cold PBS (Life Technologies). To determine the absolute number of a given cell subset per tumor, we adjusted cell counts quantitated by flow cytometry (LSR FORTESSA X-20, BD Biosciences) to absolute weight of tumor and skin from vaccine injection sites. Single-cell suspensions were prepared in PBS with 10% FCS and 2 mM EDTA (Sigma-Aldrich) by mashing tissue against the surface of a 40 μm cell strainer using the plunger of a 3 ml syringe (BD). RBCs were removed using a hypotonic lysis buffer (Stem Cell Technologies). Intracellular IFN-γ staining was performed using the Cytofix/Cytoperm kit (BD Biosciences) according to the manufacturer’s recommendation after 4 hours of stimulation with 1 μM mouse hgp100, TRP-2, or p15E peptides and using a 1:800 dilution of mAbs to IFN-γ (clone XMG1.2, BD Pharmingen). A summary of all reagents and Abs, clones, sources, and catalog numbers of reagents used for analysis can be found in Supplemental Table 2.

### Preparation of tissue homogenates.

On day 21 after tumor injection, tumor tissues were surgically removed from euthanized mice. Tissue weight measurements were performed. Tissues were homogenized in 1 ml cold PBS using a glass homogenizer. The homogenates were transferred to 1.5 ml Eppendorf tubes and centrifuged at 13,000*g* for 10 minutes at 4°C; the supernatant was stored at –80°C until analyzed by Luminex 200 (Millipore) or ELISA (Sigma-Aldrich) according to the manufacturer’s protocol. The resulting cytokine levels were then divided by the initial tumor weight for each sample.

### In vivo BLI.

C57BL/6 mice were injected in the back with 3 × 10^4^ B16 cells. Seven days later, mice were vaccinated with 100 μl of saline containing 100 μg of hgp100_25–33_ peptide s.c. and 6-day–cultured 1 × 10^6^ EGFP-sorted v-effLuc-transduced pmel-1 T cells i.v.; this was followed by 3 days of IL-2 treatment. BALB/c mice were injected i.v. with 1 × 10^6^ EGFP-sorted v-effLuc–transduced CT26 tumor. After 8 days, mice received anti–PD-L1 on days 8, 10, 12, and 14. Mice received 50 μl (1 mg kg^–1^) of 7HP349 or vehicle on days 8, 9, 10, 11, and 12. BLI was performed as previously described (15).

### Patient tissue samples.

We used RNA gene expression data from tumor tissue obtained from patients (*n* = 14) with melanoma enrolled in an ipilimumab-based sponsor-initiated clinical trial (ClinicalTrials.gov NCT02644967). Best overall response per RECIST, version 1.1, was collected for these patients.

### NanoString nCounter gene expression.

NanoString nCounter gene expression assay was performed on RNA extracted from tumor biopsies using the AllPrep DNA/RNA Kit (QIAGEN) followed by hybridization with code sets and then scanning using the nCounter Digital Analyzer per the manufacturer’s instructions (NanoString Technologies). Gene expression was analyzed using the Human PanCancer Immune Profiling Panel followed by analysis using nSolver software, version 4.0 (NanoString Technologies). nSolver was applied for quality control with default setting, and all samples passed quality control check.

### Human cell lines.

Human cell lines Jurkat, K562, HSB-2, RBL-1, 70Z/3, and DH82 were purchased from ATCC. The mutant Jurkat cell line not expressing α_4_ integrin, Jurkat (α_4_–) (35), was a gift from David Rose (UCSD, La Jolla, California, USA).

### Reagents and Abs.

Sources and catalog numbers for all reagents, recombinant proteins, cell lines, and Abs are described in Supplemental Table 2. Small molecule compounds were synthesized similarly to those described previously (14). For all assays described, compounds were dissolved in DMSO to make a series of stock solutions such that a 1:100 dilution in assay buffer or media would yield the desired final working concentrations in 1% DMSO (vehicle).

### Human T cell isolation.

Deidentified leukocyte-enriched buffy coats were purchased from the Gulf Coast Regional Blood Center (Houston, Texas, USA). The mononuclear cell fraction was enriched over a Ficoll gradient (MilliporeSigma) following standard procedures. In brief, 25 mL of cells were gently layered on top of 15 ml Ficoll. The gradient was centrifuged at 400*g* for 30 minutes with no break. The buffy coat was removed and centrifuged at 300*g* for 6 minutes with a break and the pellet resuspended in 10 mL of PBS. T cells were purified by negative selection using the MACS Pan T cell Isolation Kit (Miltenyi Biotec 130-096-535) per the manufacturer’s instructions.

CyTOF, static cell adhesion assays, LIBS epitope analysis, cell spreading assays, cell migration assays, purified α_4_β_1_-binding assays, proliferation and IL-2 production, killing assays, molecular dynamics simulations, static cell adhesion assays with CXCL12 and pertussis toxin, and TCGA analysis are described in Supplemental Methods.

### Statistics.

All results are expressed as mean ± SEM. Mouse and sample group sizes were *n* = 5, unless otherwise indicated. Data were analyzed using 1-way ANOVA, Tukey’s test, unpaired 2-tailed *t* test, or the nonparametric Kruskal-Wallis test, where applicable, and differences were considered significant at *P* < 0.05. All experiments were performed at least twice with comparable results. Mice were euthanized when the tumor size reached 200 mm^2^ or greater; survival curves were plotted using Kaplan-Meier estimates and compared by a log-rank analysis.

### Study approval.

Patient tumor samples were obtained from patients enrolled in an ipilimumab-based sponsor-initiated clinical trial (University of Texas Institutional Review Board–approved protocol, IRB 2015-0530, NCT02644967). Permission from the principal investigator of this trial was granted to use these data. All animal experiments performed in this study were approved by the Institutional Animal Care and Use Committee of The University of Texas MD Anderson Cancer Center (IACUC No. 00000745, 00000771).

## Author contributions

YH, AH, JK, TK, MCN, GW, SP, CBC, and FF conducted the investigation. YH, LS, and RJ conducted whole animal imaging and visualization procedures. LW with assistance from AH performed killing assay and analyzed the data. CH assisted with Luminex data analysis. YW and SEB assisted with patient data collection and analyzed the data. ARC, SK, NDW, RVM, RJB, DGW, and PV conducted 7HP349 in vitro characterization procedures and analyzed the data. JWC performed 7HP349 molecular dynamics simulations. YH wrote the original draft of the manuscript. WWO, DGW, PV, and MAD wrote, reviewed, and edited the manuscript. YH, WWO, DGW, and UM acquired funding. ALD, IK, UM, PH, and AD provided resources. YH supervised the study.

## Supplementary Material

Supplemental data

## Version 1 05/12/2022

In-Press Preview

## Version 2 07/01/2022

Electronic publication
