# Supplementary material for: LFA-1 activation enriches tumor-specific T cells in a cold tumor model and synergizes with CTLA-4 blockade
Source: J Clin Invest. 2022 Jul 1;132(13):e154152. doi: 10.1172/JCI154152 (PMC9246385; doi:10.1172/JCI154152)
Supplement: Supplemental data [file jci-132-154152-s232.pdf]

LFA-1 activation enriches tumor-specific T cells in a cold tumor model and synergizes with CTLA-4 blockade

Amber Hickman[1], Joost Koetsier[1], Trevin Kurtanich[1], Michael Nielsen[1], Glenn Winn[1], Yunfei Wang[1,2], Salah-Eddine Bentebibel[1], Leilei Shi[1], Simone Punt[1], Leila Williams[1], Cara Haymaker[3], Charles B. Chesson[1], Faisal Fa'ak[1], Ana Dominguez[1,2], Richard Jones[4], Isere Kuiatse[4], Amy R. Caivano[5], Sayadeth Khounlo[5], Navin D. Warier[5], Upendra Marathi[6], Robert V. Market[5], Ronald J. Biediger[5], John W. Craft, Jr.[7], Patrick Hwu[1,2], Michael A. Davies[1], Darren G. Woodside[5], Peter Vanderslice[5], Adi Diab[1], Willem W. Overwijk[1,8], and Yared Hailemichael[1,9,*]

[1]Department of Melanoma Medical Oncology, The University of Texas MD Anderson Cancer Center, Houston, Texas, USA. [2] Present address Moffitt Cancer Center, Tampa, Florida, USA. [3]Department of Translational Molecular Pathology, The University of Texas MD Anderson Cancer Center, Houston, Texas, USA. [4]Department of Lymphoma Myeloma, The University of Texas MD Anderson Cancer Center Houston, Texas, USA. [5]Molecular Cardiology Research Laboratories, Texas Heart Institute, Houston, Texas, USA. [6]7 Hills Pharma, Houston, Texas, USA. [7]Department of Biology and Chemistry, University of Houston, Texas, USA. [8]Present address Nektar Therapeutics, San Francisco, California, USA.

[9]Lead Contact

*Correspondence:

Yared Hailemichael

Melanoma Medical Oncology

The UT MD Anderson Cancer Center

7455 Fannin Street, Unit 0904

Houston, Texas, 77054, USA

Ph# (713) 792-1931

yhailemi@mdanderson.org

Supplemental Material

1. Supplemental Figures (1 - 16)
2. Supplemental Methods
3. Supplemental Table 1
4. Supplemental Table 2

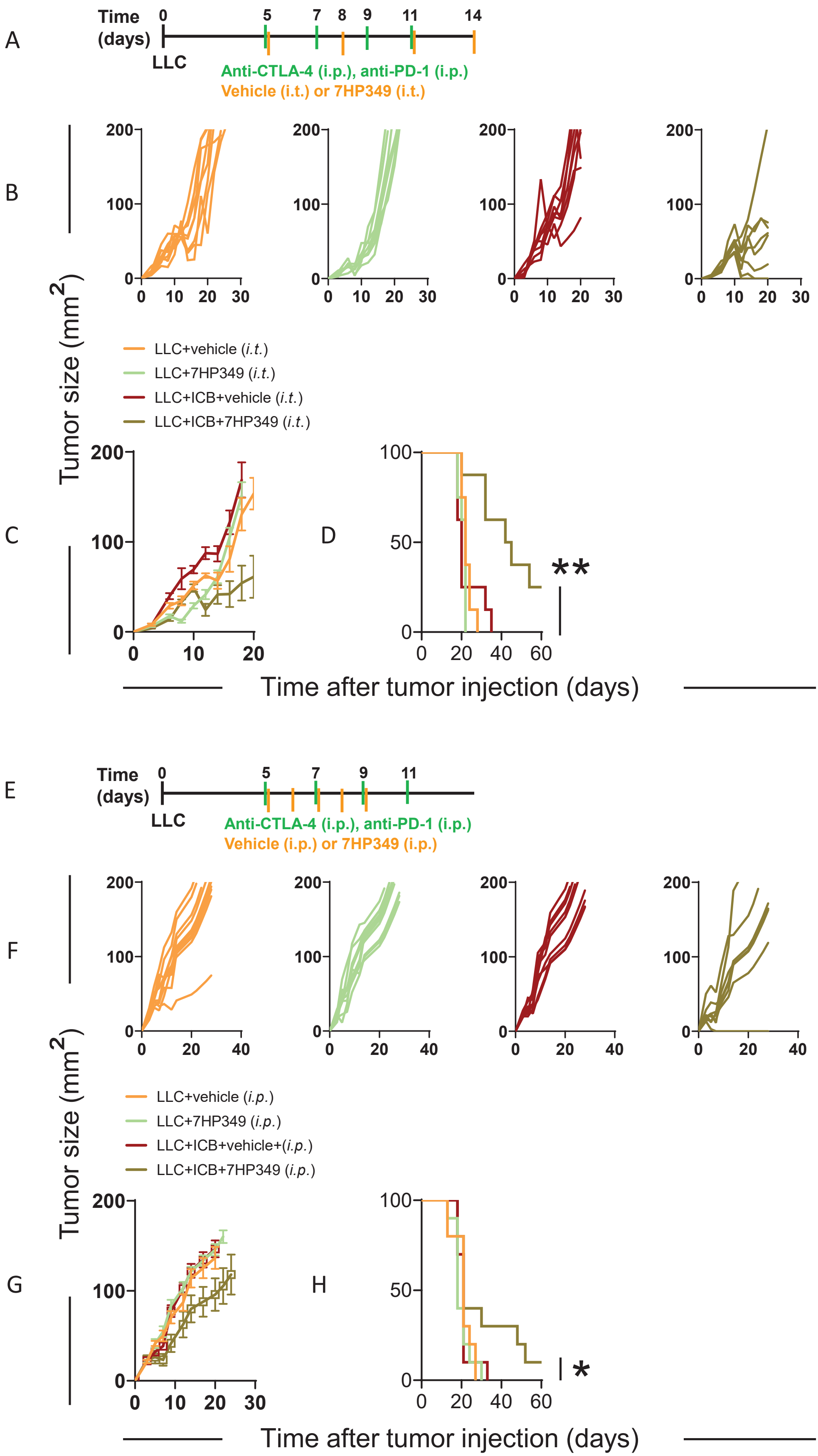
A
Time (days)
0
5
7
8
9
11
14
LLC
Anti-CTLA-4 (i.p.), anti-PD-1 (i.p.)
Vehicle (i.t.) or 7HP349 (i.t.)
B
Tumor size (mm2)
LLC+vehicle (i.t.)
LLC+7HP349 (i.t.)
LLC+ICB+vehicle (i.t.)
LLC+ICB+7HP349 (i.t.)
C
D
**
Time after tumor injection (days)
E
Time (days)
0
5
7
9
11
LLC
Anti-CTLA-4 (i.p.), anti-PD-1 (i.p.)
Vehicle (i.p.) or 7HP349 (i.p.)
F
Tumor size (mm2)
LLC+vehicle (i.p.)
LLC+7HP349 (i.p.)
LLC+ICB+vehicle+(i.p.)
LLC+ICB+7HP349 (i.p.)
G
H
*
Time after tumor injection (days)

Supplemental Figure 1. 7HP349 therapy overcomes primary resistance to combination checkpoint blockade.
(A-D) C57BL/6 mice received anti-CTLA-4 (i.p.) and anti-PD-1 (i.p.) 5 days after LLC tumor injection and/or vehicle intratumorally (i.t.) or 7HP349 (i.t.) 2 weeks (2 x weekly) after tumor injection, as indicated: (A) Treatment scheme, (B) Tumor growth curves (C) Average tumor burden (mean ± SEM, $n = 10$, $^{*}P < 0.05$, $^{**}P < 0.01$ one-way ANOVA), and Kaplan-Meier survival curve ($n = 8$) $^{**}P < 0.001$ log-rank test.
(E-H) Mice were treated as in 'A' and but with systemic intraperitoneal dosing of 7HP349 or vehicle 5 days after tumor injection 1 week (5 x weekly): (E) experimental scheme, (F) individual tumor burden plots, (G) (mean ± SEM., $n = 10$, $^{*}P < 0.05$, one-way ANOVA), and (H) Kaplan-Meier survival curve ($n = 8$) $^{*}P < 0.05$ log-rank test.

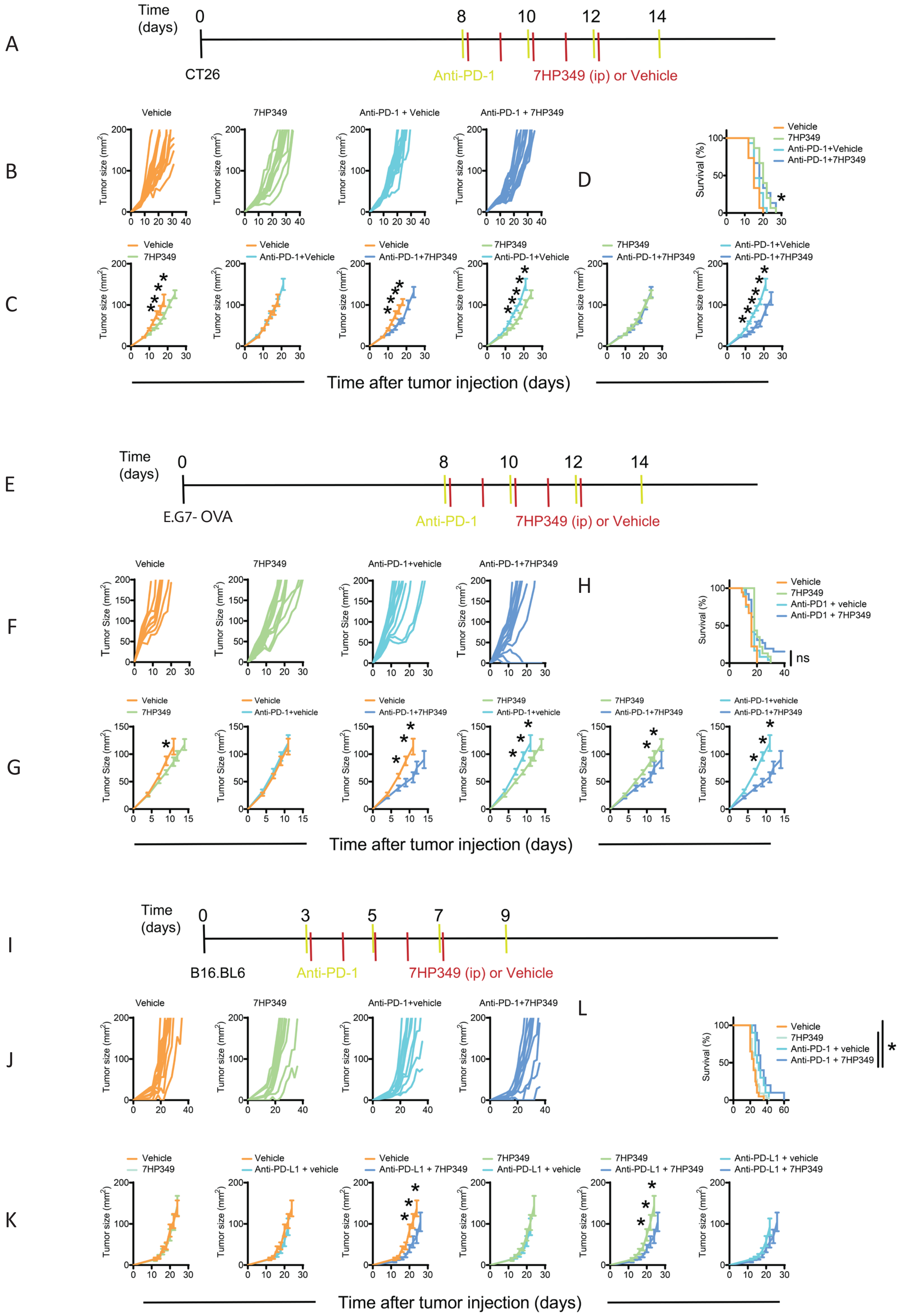
A
Time (days)
0
8
10
12
14
CT26
Anti-PD-1
7HP349 (ip) or Vehicle
B
Vehicle
7HP349
Anti-PD-1 + Vehicle
Anti-PD-1 + 7HP349
Tumor size (mm2)
D
Survival (%)
Vehicle
7HP349
Anti-PD-1+Vehicle
Anti-PD-1+7HP349
C
Time after tumor injection (days)
E
Time (days)
E.G7- OVA
Anti-PD-1
7HP349 (ip) or Vehicle
F
Vehicle
7HP349
Anti-PD-1+vehicle
Anti-PD-1+7HP349
Tumor Size (mm2)
H
Anti-PD1 + vehicle
Anti-PD1 + 7HP349
ns
G
Time after tumor injection (days)
I
Time (days)
0
3
5
7
9
B16.BL6
Anti-PD-1
7HP349 (ip) or Vehicle
J
L
Anti-PD-1 + vehicle
Anti-PD-1 + 7HP349
K
Anti-PD-L1 + vehicle
Anti-PD-L1 + 7HP349
Time after tumor injection (days)

Supplemental Figure 2. 7HP349 therapy overcomes primary resistance to PD-1 checkpoint blockade.

(A-D) Balb/c mice 8 days after subcutaneous injection with CT26 cells received 7HP349 or vehicle intraperitoneally (i.p.), at days 8, 9, 10, 11 and 12 with anti-PD-1 at days 8, 10, 12 and 14. Tumors were harvested 21 days after injection. (A) Treatment scheme. (B-D) Tumor burden measurement with anti-PD-1 (B) Tumor growth curves (C) Average tumor burden (mean ± SEM, $n = 10$) one-way ANOVA, Tukey test. $^*P < 0.05$. (D) Kaplan-Meier survival curve (n = 10), $^*p < 0.05$ log-rank test.

(E-H) C57BL/6 mice 8 days after s.c. injection with E.G-OVA 7 cells received 7HP349 or vehicle intraperitoneally (i.p.) at days 8, 9, 10, 11 and 12 with anti-PD-1 (i.p.) at days 8, 10, 12 and 14. (E) Treatment scheme. (F) Tumor growth curves (G) Average tumor burden (mean ± SEM, $n = 10$) one-way ANOVA, Tukey test. $^*P < 0.05$. (H) Kaplan-Meier survival curve ($n = 10$), $^*P < 0.05$, log-rank test.

(I-L) C57BL/6 mice 3 days after s.c. injection with B16.BL6 cells received 7HP349 or vehicle intraperitoneally (i.p.), at days 3, 4, 5, 6 and 7 with anti-PD-1 (i.p.) at days 3, 5, 7 and 9 (I) Experimental scheme. Tumor burden measurement (J) Tumor growth curves (K) mean ± SEM ($n = 10$) one-way ANOVA, Tukey test. $^*P < 0.05$. (L) Kaplan-Meier survival curve ($n = 10$), $^*P < 0.05$, log-rank test.

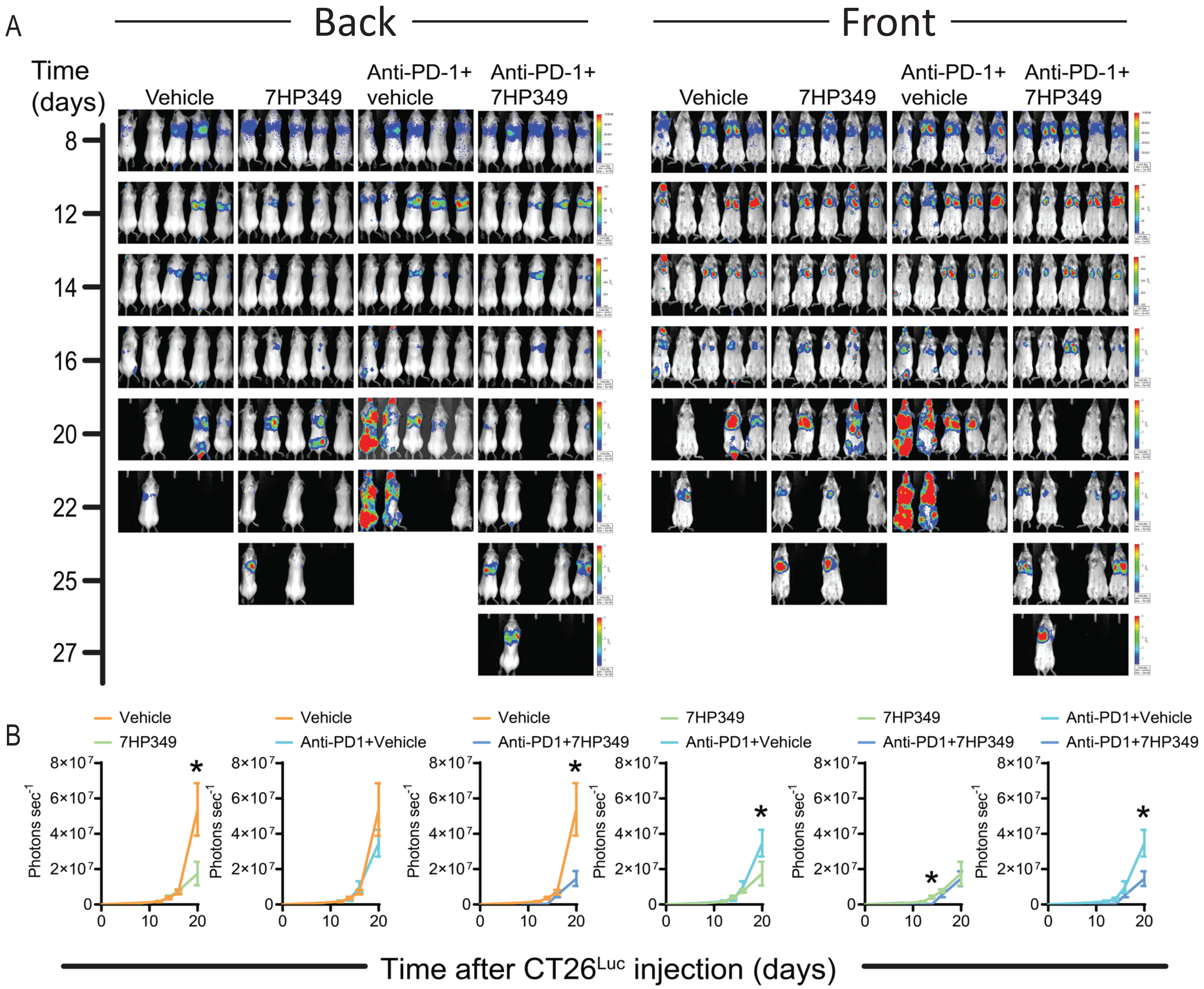

Supplemental Figure 3. 7HP349 therapy overcomes primary resistance to PD-1 blockade in pulmonary metastatic tumor model.
(A, B) Balb/c mice received 1x10^{6} luciferase-expressing CT26 cells i.v. Mice received vehicle, 7HP349, anti-PD-1, or the combination at the indicated timepoints, asin (A). Luciferase-expressing CT26 cells werevisualized by whole body imaging at the indicated time points. (B) lLine plots represent means ± SEM. photons /second (S^{-1}) (*n*=5, **P* < 0.05, unpaired t test).

A

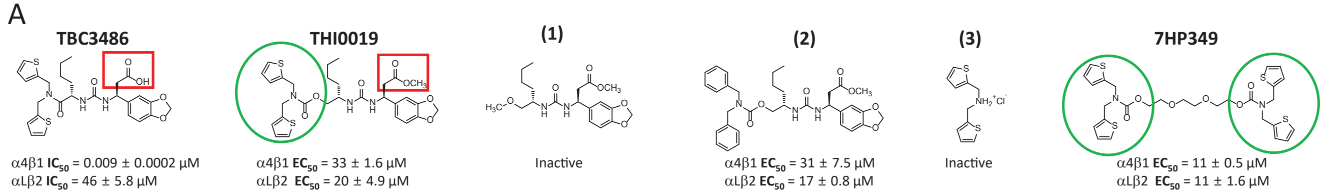

B

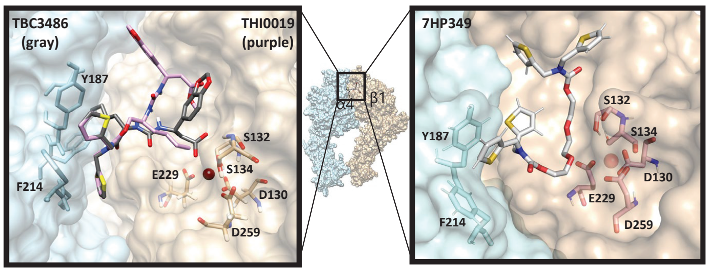

C

D

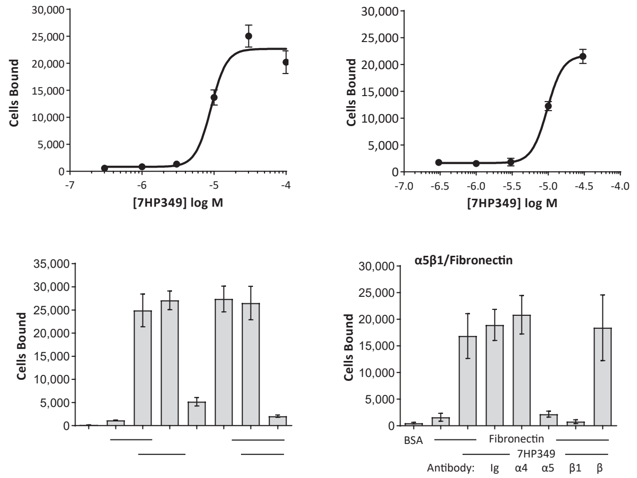

E

Integrin	Ligand	EC_{50} (μM)
α4β1	VCAM-1	11 ± 0.5
αLβ2	ICAM-1	11 ± 1.6
α4β7	MAdCAM-1	10 ± 2.1
α5β1	Fibronectin	7.7 ± 2.3
α1β1	Collagen IV	not active
α2β1	Collagen I	not active
αVβ3	Vitronectin	not active
αIIbβ3	Fibrinogen	not active

F

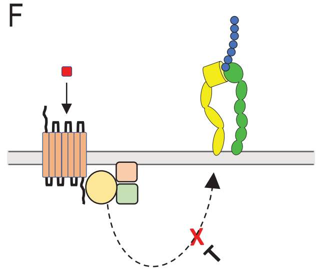

G

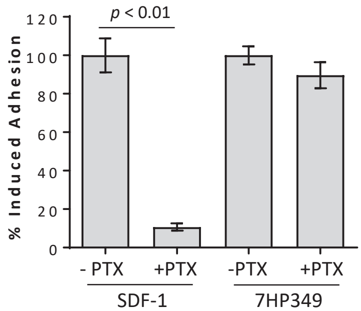

H

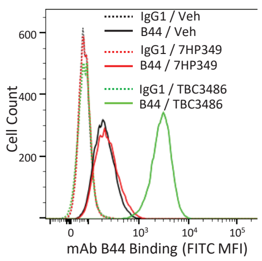

LIBS mAb	Treatment	MFI (IgG)	MFI (mAb)
B44	Veh	87 ± 7.8	330 ± 82
	7HP349	104 ± 13	326 ± 92^{ns}
	TBC3486	96 ± 8.1	2448 ± 280*
HUTS-21	Veh	89 ± 10	294 ± 87
	7HP349	95 ± 15	201 ± 49^{ns}
	TBC3486	89 ± 14	2112 ± 492*
9EG7	Veh	70 ± 2.2	294 ± 58
	7HP349	72 ± 2.7	215 ± 22^{ns}
	TBC3486	72 ± 3.0	722 ± 85*

I

Species (cell line)	EC_{50} (μM)
Human (Jurkat)	2.0 ± 0.8
Mouse (70Z/3)	2.0 ± 0.8
Rat (RBL-1)	2.6 ± 0.8
Dog (DH82)	2.0 ± 0.1

Supplemental Figure 4. Integrin activator 7HP349.
(A) Compound structures.
(B) Molecular docking of integrin activators into a model of integrin α4β1.
(C) K562 cells expressing integrin α4β7 adhesion to plastic immobilized MAdCAM-1.
(D) K562 cell adhesion to plastic immobilized fibronectin.
(E) 7HP349 selectivity and EC50's of 7HP349 against various integrin targets (mean ± s.d., $n = 3$).
(F,G) 7HP349 induced cell adhesion is not sensitive to Pertussis Toxin (PTX) treatment
(F) Cartoon schematic of GPCR signaling (G) Jurkat cell adhesion to immobilized α4β1 ligand CS-1 (mean ± s.d., $n = 3$).
(H) 7HP349 cross-species activity against integrin α4β1 (mean ± s.d., $n = 3$).
(I) 7HP349 does not induce exposure of β1 integrin LIBS epitopes (mean fluorescence intensity (MFI) ± SEM, $n = 3$, $^{**}P < 0.01$).

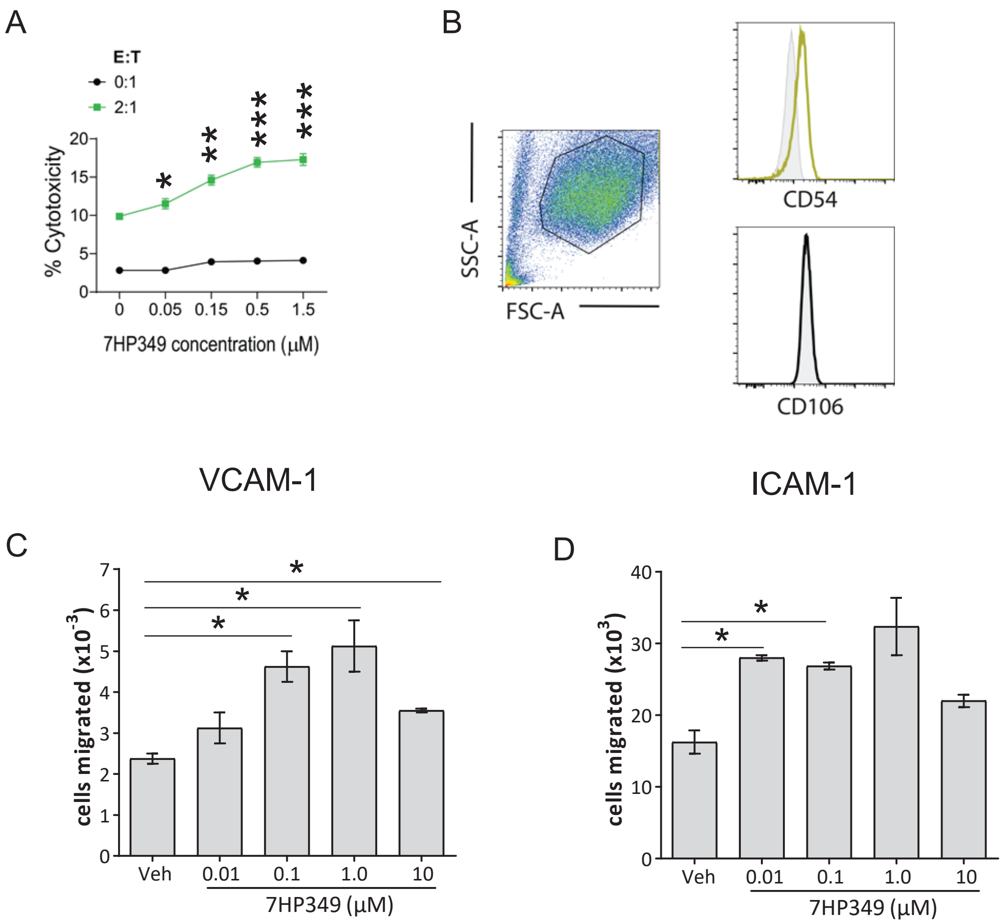

Supplemental Figure 5. 7HP349 enhances cytolytic activity and cell migration. (A) Effector T cells (TIL) from patient derived melanoma cell lines were incubated with HLA-matched target tumor cell line 2767 at the indicated effector / target (E/T) ratios and 7HP349 dose concentrations for 3 hours. Cell death, as determined by caspase-3–positive cells (means ± SEM, *n* = 3, * $P \leq 0.05$; ** $P \leq 0.01$, *** $P \leq 0.001$, unpaired two-tailed t test).
(B) Flow cytometer analysis showing ICAM-1 (CD54) and VCAM-1 (CD106) expressions on 2767 tumor cell line.
(C,D) 7HP349 dose-dependent enhancement of Jurkat cell CXCL12 dependent chemotaxis on α4β1 and αLβ2 ligands (D) VCAM-1 (mean ± s.d., *n* = 3, * $P \leq 0.05$; Tukey test) (E) ICAM-1 (means ± s.d., *n* = 3, * $P \leq 0.05$; Tukey test).

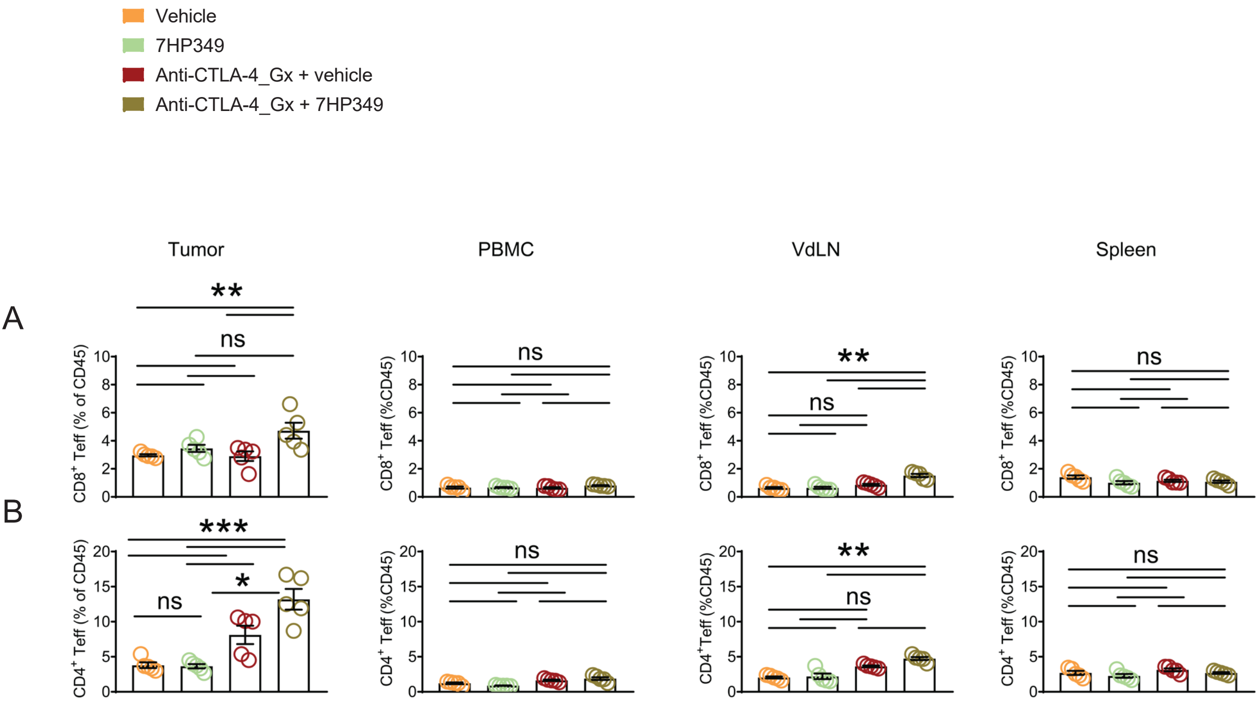

Supplemental Figure 6. 7HP349 treatment enhances T cell localization to tumor. C57BL/6 mice 3 days after s.c.injection with B16.BL6 cells received 7HP349 or vehicle intraperitoneally (i.p.), at days 3, 4, 5, 6 and 7 with GVAX intradermally (i.d.) and anti-CTLA-4 intraperitoneally (i.p.) at days 3, 6 and 9. Tumors were harvested on d 21.

(A) $CD8^+$ Teffl evel (% of CD45) in tumor, PBMC, VdLN and Spleen means ±s.e.m. (n = 5, *p < 0.05, one-way ANOVA).

(B) $CD4^+$ Teff level (% of CD45) in tumor, PBMC, VdLNand Spleen means ±s.e.m. (*n* = 5, **P* < 0.05, one-way ANOVA).

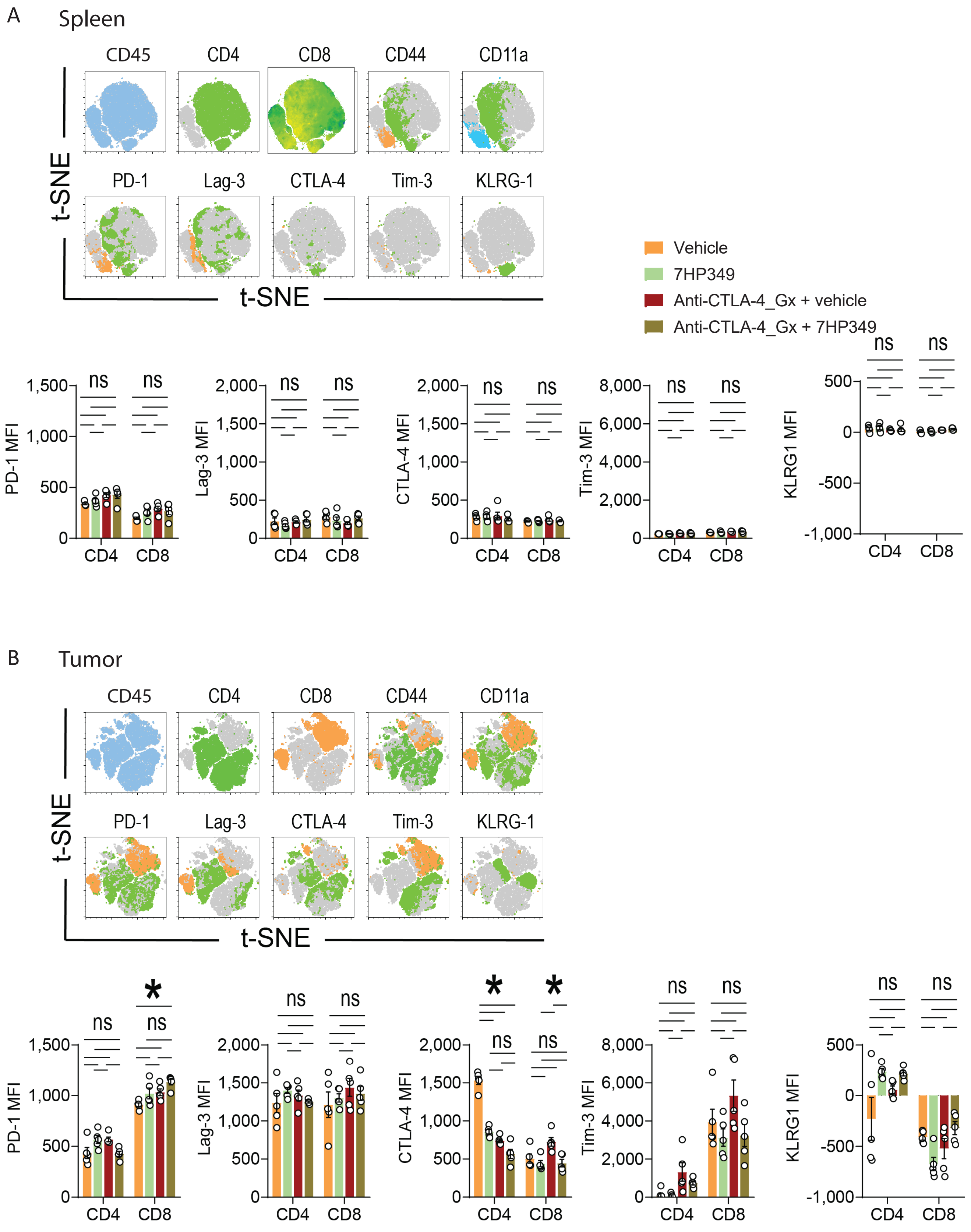
A
Spleen
CD45
CD4
CD8
CD44
CD11a
PD-1
Lag-3
CTLA-4
Tim-3
KLRG-1
t-SNE
t-SNE
Vehicle
7HP349
Anti-CTLA-4_Gx + vehicle
Anti-CTLA-4_Gx + 7HP349
PD-1 MFI
Lag-3 MFI
CTLA-4 MFI
Tim-3 MFI
KLRG1 MFI
ns
CD4
CD8
B
Tumor
CD45
CD4
CD8
CD44
CD11a
PD-1
Lag-3
CTLA-4
Tim-3
KLRG-1
t-SNE
t-SNE
PD-1 MFI
Lag-3 MFI
CTLA-4 MFI
Tim-3 MFI
KLRG1 MFI
ns
*
CD4
CD8

Supplemental Figure 7. Activation markers expression on spleen and tumor infiltrating $CD8^+$ and $CD4^+$ T effectors.

C57BL/6 mice 3 days after s.c.injection with 3×104B16.BL6 cells received 7HP349 or vehicle intraperitoneally(i.p.), at days 3, 4, 5, 6 and 7 with GVAX intradermally(i.d.) and anti-CTLA-4 intraperitoneally(i.p.) at days 3, 6 and 9. Spleen and tumor tissues were harvested at d 21. t-SNE dimension reduction plots comparing activation marker expression on $CD4^+$ and $CD8^+$ T cells in (A) spleen (mean ± SEM, n = 5) (B) tumor (mean ± SEM, n = 5, *$P < 0.05$, one-way ANOVA, Tukey test).

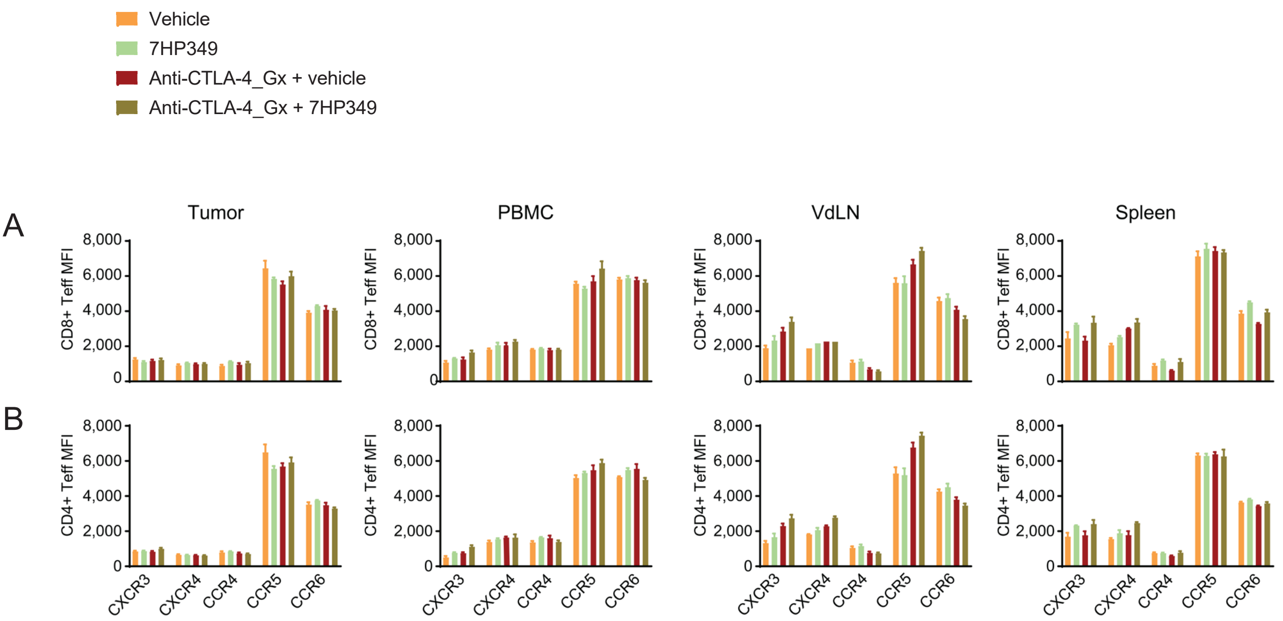

Supplemental Figure 8. CD8$^+$ and CD4$^+$ Teff chemokine receptor expression. C57BL/6 mice 3 days after s.c.injection with B16.BL6 cells received 7HP349 or vehicle intraperitoneally (i.p.), at days 3, 4, 5, 6 and 7 with GVAX intradermally (i.d.) and anti-CTLA-4 intraperitoneally(i.p.) at days 3, 6 and 9. Tumors were harvested on d 21.

(A) CD8$^+$ Teff chemokine receptor expression (MFI) in tumor, PBMC, VdLN and spleen mean ± SEM. (n = 5).

(B) CD4$^+$ Teff chemokine receptor expression (MFI) in tumor, PBMC, VdLN and spleen means ± SEM (n = 5).

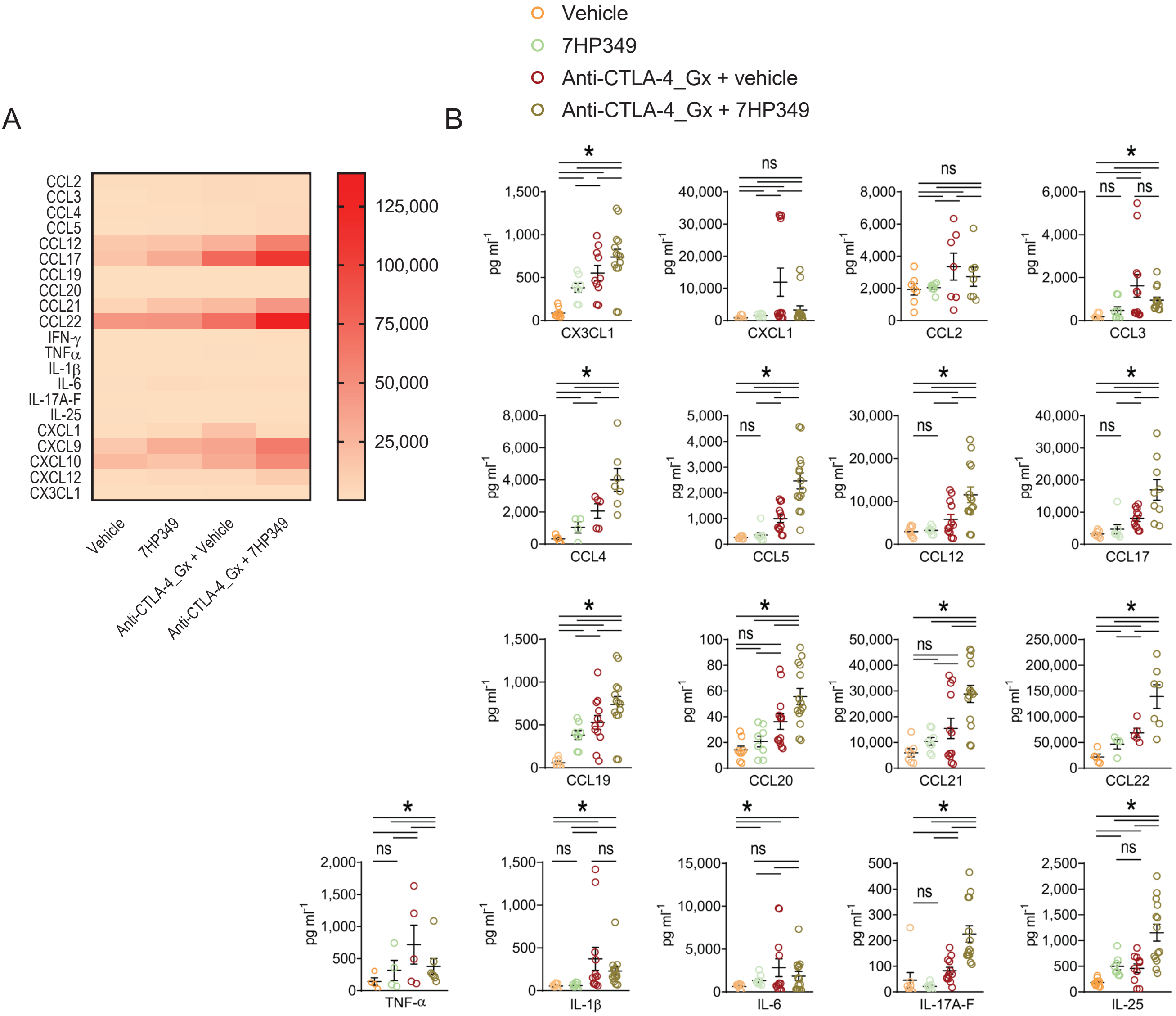

Supplemental Figure. 9. Proinflammatory cytokine expression in tumor.

C57BL/6 mice 3 days after s.c.injection with $3×10^4$ B16.BL6 cells received 7HP349 or vehicle intraperitoneally(i.p.), at days 3, 4, 5, 6 and 7 with GVAX intradermally(i.d.) and anti-CTLA-4 intraperitoneally(i.p.) at days 3, 6, and 9. Tumors were harvested on d 21. (A) Heat map displaying normalized cytokine and chemokine expression across treatment groups (B) concentration (means ±s.e.m., *n* = 5, **P* < 0.05, one-way ANOVA).

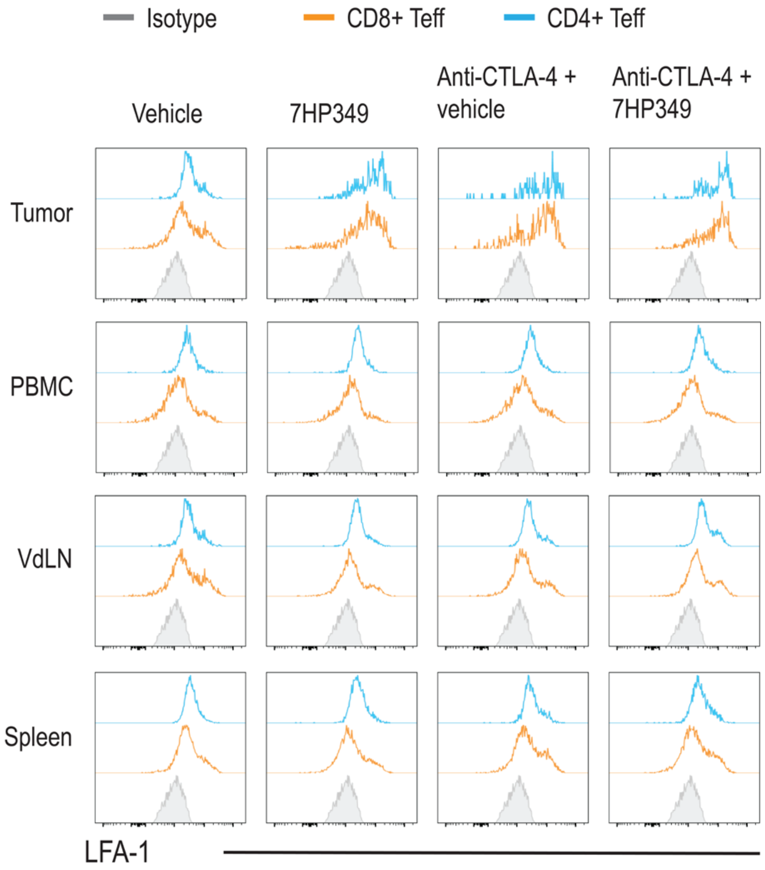

Supplemental Figure 10. Flow cytometer histogram panels showing LFA-1 expression on CD4^{+} and CD8^{+} Teff from tumor, PBMC, lymph node and spleen.

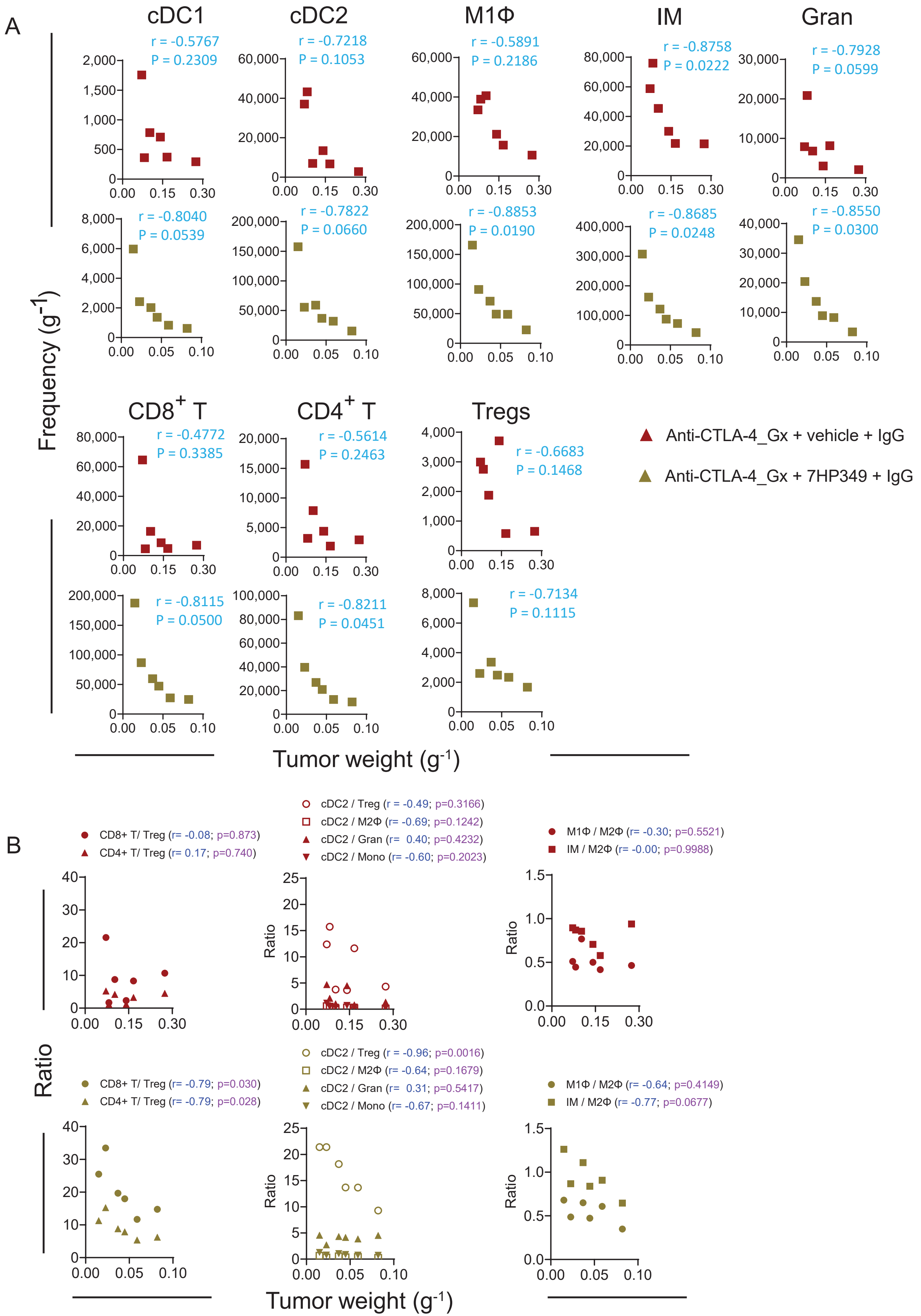
A
cDC1
cDC2
M1Φ
IM
Gran
r = -0.5767
P = 0.2309
r = -0.7218
P = 0.1053
r = -0.5891
P = 0.2186
r = -0.8758
P = 0.0222
r = -0.7928
P = 0.0599
r = -0.8040
P = 0.0539
r = -0.7822
P = 0.0660
r = -0.8853
P = 0.0190
r = -0.8685
P = 0.0248
r = -0.8550
P = 0.0300
Frequency (g-1)
CD8+ T
CD4+ T
Tregs
r = -0.4772
P = 0.3385
r = -0.5614
P = 0.2463
r = -0.6683
P = 0.1468
r = -0.8115
P = 0.0500
r = -0.8211
P = 0.0451
r = -0.7134
P = 0.1115
Anti-CTLA-4_Gx + vehicle + IgG
Anti-CTLA-4_Gx + 7HP349 + IgG
Tumor weight (g-1)
B
CD8+ T/ Treg (r= -0.08; p=0.873)
CD4+ T/ Treg (r= 0.17; p=0.740)
cDC2 / Treg (r = -0.49; p=0.3166)
cDC2 / M2Φ (r= -0.69; p=0.1242)
cDC2 / Gran (r= 0.40; p=0.4232)
cDC2 / Mono (r= -0.60; p=0.2023)
M1Φ / M2Φ (r= -0.30; p=0.5521)
IM / M2Φ (r= -0.00; p=0.9988)
Ratio
CD8+ T/ Treg (r= -0.79; p=0.030)
CD4+ T/ Treg (r= -0.79; p=0.028)
cDC2 / Treg (r = -0.96; p=0.0016)
cDC2 / M2Φ (r= -0.64; p=0.1679)
cDC2 / Gran (r= 0.31; p=0.5417)
cDC2 / Mono (r= -0.67; p=0.1411)
M1Φ / M2Φ (r= -0.64; p=0.4149)
IM / M2Φ (r= -0.77; p=0.0677)
Tumor weight (g-1)

Supplemental Figure 11. Correlation analysis between tumor weight and immune cell infiltrates.

C57BL/6 mice 3 days after s.c.injection with BL6 cells received 7HP349 or vehicle intraperitoneally (i.p.), at days 3, 4, 5, 6 and 7 with GVAX intradermally(i.d.) and anti-CTLA-4 intraperitoneally (i.p.) at days 3, 6, and 9. Tumors were harvested on d 21. Correlation analysis between tumor weight and (A) myeloid cell frequency (g^{-1}) (B) immune cell ratios.

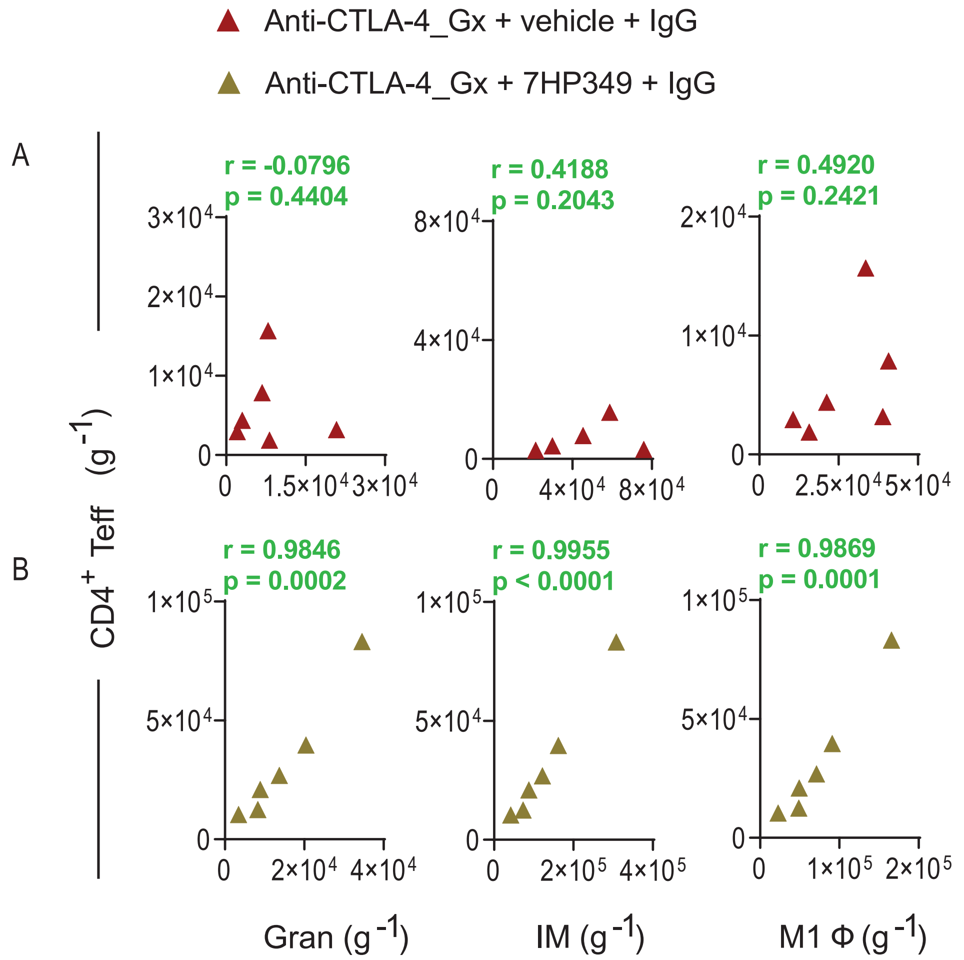

Supplemental Figure 12. Correlation analysis between myeloid and CD4$^+$ B16 tumor immune cell infiltrates.

Mice bearing 3-day s.c.B16.BL6 melanomas received GVAX intradermally(i.d.) and anti-CTLA-4 (i.p.) at days 3, 6, and 9 and vehicle or 7HP349 at days 3, 4, 5, 6, and 7.

Correlation analysis of granulocytes, IM, or M1Φ versus CD4$^+$ Teff from tumors of mice treated with (A) vehicle (B) 7HP349.

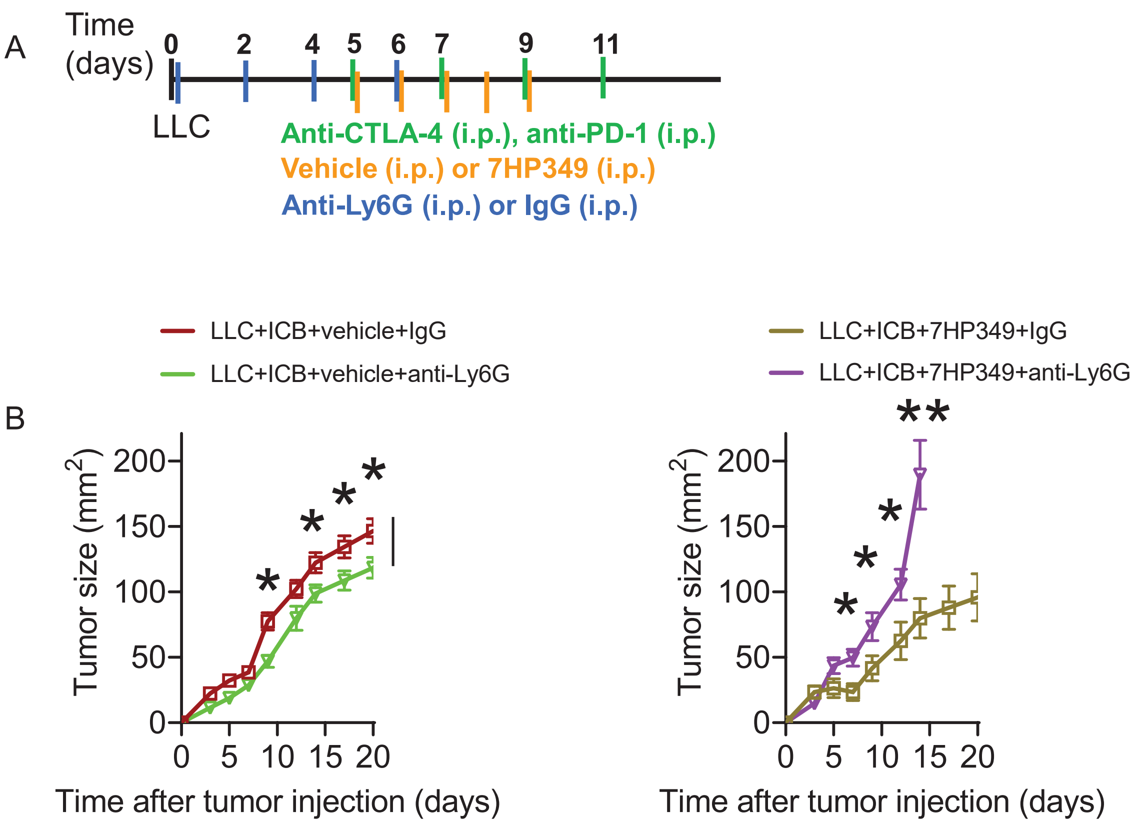

Supplemental Figure 13. 7HP349 antitumor effect is dependent on neutrophils. C57BL/6 mice injected with LLC tumor were treated with anti-CTLA-4, anti-PD-1 (IBC), 7HP349 and/or vehicle and anti-Ly6G or IgG, as indicated.

(A) Treatment scheme. (B) Average tumor burden in mice (n = 10). Data are represented as mean ± SEM, one-way ANOVA, Tukey test, * $P < 0.05$, ** $P < 0.01$.

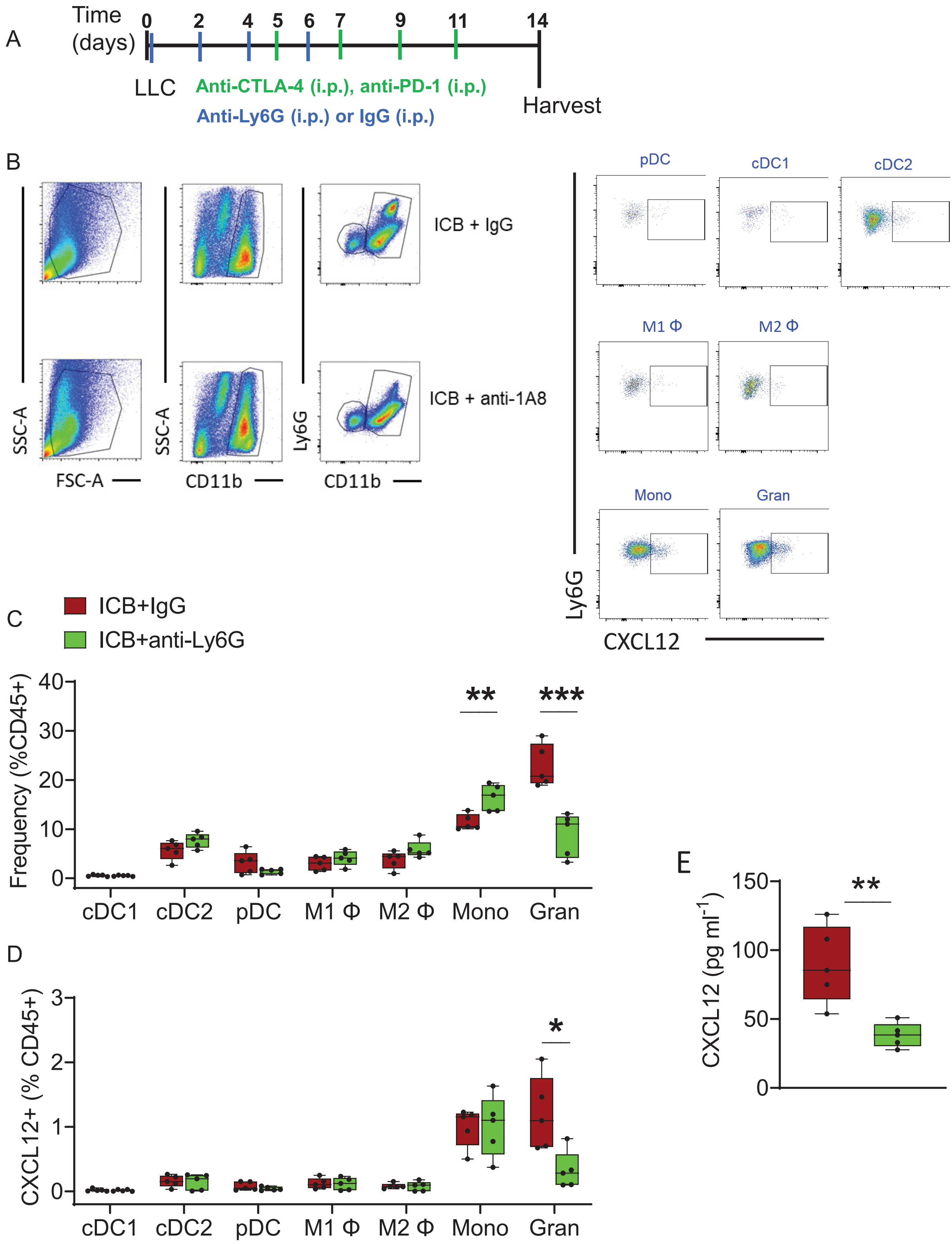
A
Time (days)
0
2
4
5
6
7
9
11
14
LLC
Anti-CTLA-4 (i.p.), anti-PD-1 (i.p.)
Anti-Ly6G (i.p.) or IgG (i.p.)
Harvest
B
SSC-A
FSC-A
SSC-A
CD11b
Ly6G
CD11b
ICB + IgG
ICB + anti-1A8
pDC
cDC1
cDC2
M1 Φ
M2 Φ
Mono
Gran
Ly6G
CXCL12
C
ICB+IgG
ICB+anti-Ly6G
Frequency (%CD45+)
cDC1
cDC2
pDC
M1 Φ
M2 Φ
Mono
Gran
D
CXCL12+ (% CD45+)
E
CXCL12 (pg ml-1)

Supplemental Figure 14. Neutrophils are main source of CXCL12 at the TME. C57BL/6 mice received anti-CTLA-4 (i.p.) and anti-PD-1 (i.p.) 5 days after LLC tumor injection and IgG (i.p.) or anti-Ly6G (i.p.). Tumors were harvested 14 days after injection. (A) Experimental scheme (B) Flow cytometry analysis showing CXCL12 secretion by myeloid cell populations in tumor (C-E) Combination of box-and-whisker and dot plots showing (C) the total cDC1, cDC2, pDC, M1, M2, monocytes and granulocytes as percentage of $CD45^+$ leukocytes. (D) $CXCL12^+$ cDC1, cDC2, pDC, M1 macrophage, M2 macrophage, monocytes and granulocytes as percentage of $CD45^+$ leukocytes. (E) CXCL12 concentration in tumor supernatant analyzed by ELISA. Data are represented as (mean ± SEM, n = 5). The whiskers are the minimum and maximum values, the lower and upper box edges the 25th and 75th percentage values, respectively, and the lines within the boxes the median. Data were analyzed using unpaired t test. *P < 0.05, **P < 0.01, ***P < 0.001.

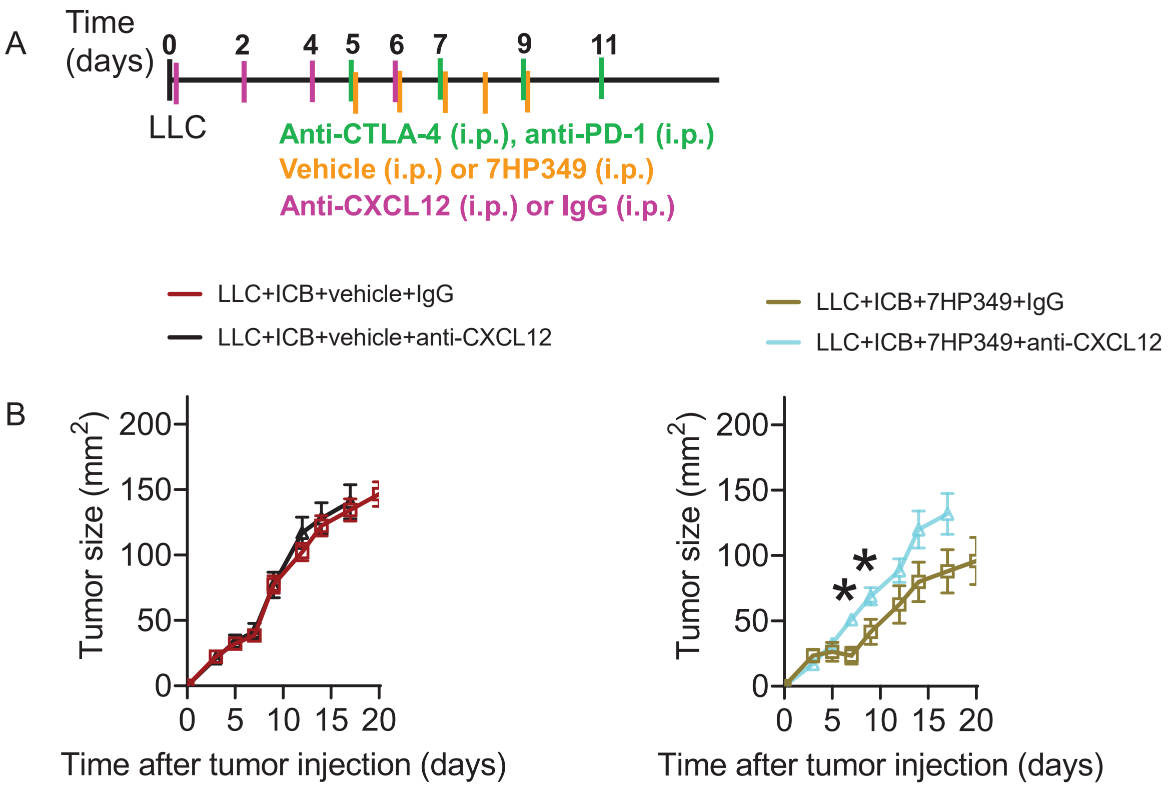

Supplemental Figure 15. 7HP349 antitumor effect is dependent on CXCL12. C57BL/6 mice injected with LLC tumor were treated with anti-CTLA-4, anti-PD-1 (IBC), 7HP349 and/or vehicle and anti-CXCL12 or IgG, as indicated.

(A) Treatment scheme. (B) Average tumor burden in mice (n = 10). Data are represented as mean ± SEM, one-way ANOVA, Tukey test, ** $P < 0.01$.

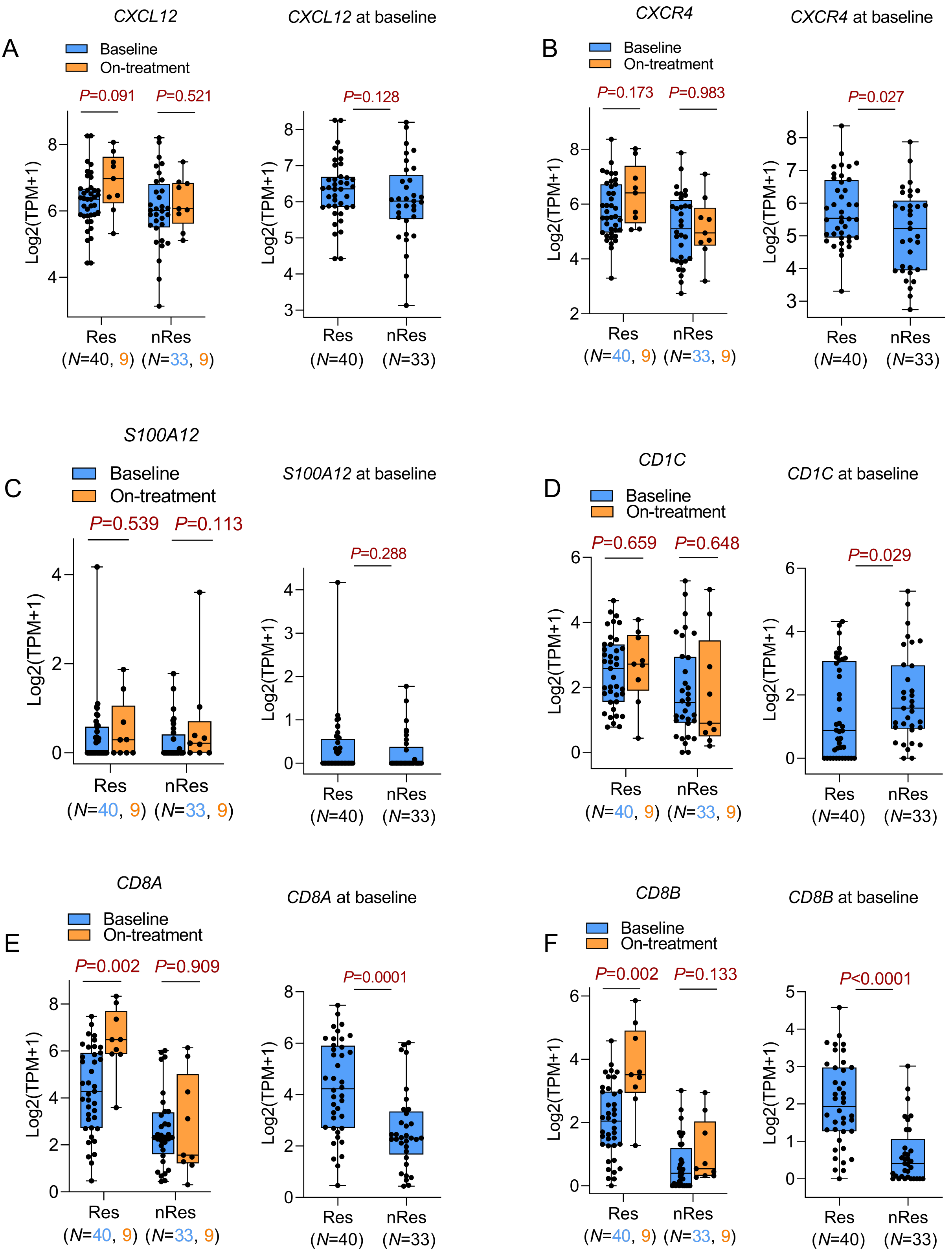

A
CXCL12
Baseline
On-treatment
P=0.091
P=0.521
Log2(TPM+1)
Res
nRes
(N=40, 9)
(N=33, 9)
CXCL12 at baseline
P=0.128
(N=40)
(N=33)
B
CXCR4
P=0.173
P=0.983
CXCR4 at baseline
P=0.027
C
S100A12
P=0.539
P=0.113
S100A12 at baseline
P=0.288
D
CD1C
P=0.659
P=0.648
CD1C at baseline
P=0.029
E
CD8A
P=0.002
P=0.909
CD8A at baseline
P=0.0001
F
CD8B
P=0.002
P=0.133
CD8B at baseline
P<0.0001

Supplemental Figure 16. CXCR4 gene expression signature predict response to CTLA-4 checkpoint blockade in melanoma.

RNASeq gene expression level (log2-transformed TPM with pseudocount 1) difference between responder and non-responder groups in metastatic melanoma patients. Individual gene expression changes between baseline and on treatment with anti-PD-1 or anti-CTLA-4 + anti-PD-1. Boxplots show differentially expressed CXCL12, CXCR4, CD1C, S100A12, CD8A, CD8B mRNA level from pooled data of patients responding (Res) and non-responding (nRes) to treatment (* $p < 0.05$, ** $p < 0.01$, *** $p < 0.001$, **** $p < 0.0001$) as determined using the limma-trend method. Data are presented as median and whiskers on the box plots extend minimum to maximum points. The top and bottom lines of the box plots represent the interquartile range (IQR), the mid line represents the median, and the whiskers on the box plots represent minimum and maximum values.

Supplemental Methods

CyTOF. The CyTOF assay was performed according to the manufacturer's instructions. In brief, 3 × 10^6 cells were stained with 5 mM Cell-ID cisplatin (Fludigm) for 5 minutes and quenched with MaxPar Cell Staining Buffer (Fludigm) using 5 times the volume of the cell suspension. After centrifugation, cell suspensions (50 μl) were incubated with 5 μL of human Fc-receptor Blocking solution for 10 minutes and 50 μL of pre-mixed antibody cocktail for 30 minutes. After being washed, cells were incubated with 1 ml of cell intercalation solution 125 nM MaxPar Intercalator-Ir (Fludigm) into 1 ml MaxPar Fix and Perm Buffer (Fludigm) overnight at 4°C. Cells were centrifuged with MaxPar Water and pelleted. The pelleted cells were suspended with EQ Calibration Beads, and cell events were acquired by a Helios CyTOF mass cytometer. A summary of all mass cytometry reagents and antibodies, and their clone, source and catalogue #s used for analysis can be found in (Supplemental Table 2).

Static cell adhesion assays. Ligands (VCAM-1, ICAM-1, MAdCAM-1, fibronectin, fibrinogen, collagen I, and IV) in 50 μL of 50 mM Tris-HCl (pH 7.4), 150 mM NaCl, TBS were added to wells of a 96-well plate and allowed to coat overnight at 4°C. To maximize the window to evaluate agonist activity, a sub-optimal coating concentration of ligand was used. This ligand concentration corresponded approximately to that which would yield ≤ 5% adhesion as determined by dose-response curves of ligand binding to the appropriate cell type. Unless otherwise stated in figure legends, the concentrations of ligands used for the assays were 0.5 μg/mL VCAM-1, 5 μg/mL ICAM-1, 1 μg/mL MAdCAM-1, 1 μg/mL fibronectin, 1 μg/mL Collagen I, 0.3 μg/mL Collagen IV, and 3 μg/mL fibrinogen. All assays were performed as previously described(14). Briefly, 2 x 10^6 cells were labeled for 30 minutes with calcein-AM (Thermo Fisher Scientific), washed, resuspended in binding buffer, and added to triplicate wells of ligand-coated

plates (2 x 10^5 cells/well) that had been blocked with 2% BSA. After a 30-minute incubation at 37°C, the plates were washed 3 times with binding buffer, the adherent cells were lysed, and fluorescence was measured on a Tecan Safire 2 plate reader (TECAN). Standard curves were run for each assay to convert fluorescence units directly to cell number.

LIBS epitope analysis. Jurkat cells were suspended in 100μL buffer (Tyrode's containing 1 mg/ml glucose and 1mM $MgCl_2$, 1 mM $CaCl_2$). Primary LIBS mAb B44, HUTS-21, or 9EG7 (10 μg/ml) was added, and cells were incubated for 1 hour on ice. Vehicle, 10 μM TBC3486, or 10μM 7HP349 was added at the same time as primary mAb. Cells were washed and resuspended in 50μL buffer containing FITC-conjugated GAM secondary antibody and were then incubated on ice for 30 minutes. After another 3 washes with buffer, cells were resuspended in 500μL buffer and were analyzed on a Beckman Coulter Epics XL-MCL (GMI). MFI was determined from 3 separate experiments, each performed on a different day.

Cell spreading assays. Human VCAM-1 (50 μl of 4 μg/ml) was coated in TBS overnight at 4°C onto 96-well plates (Costar). Plates were blocked with 2% BSA for 1 hour at room temperature and were washed with complete media (RPMI-1640 supplemented with 10% FBS, 100 units/ml penicillin, and 100 μg/ml streptomycin). HSB cells (10^4 cells in complete media) were mixed with vehicle or 10 μM 7HP349 immediately prior to adding to the wells and incubated for the indicated time points at 37°C. Images of cells were captured at 10X magnification on an Olympus IX71 inverted microscope (Olympus America, Inc., Center Valley, PA) equipped with an AHHO39020 CCD camera. For each treatment group, images were analyzed in a blinded fashion using NIH ImageJ software (https://imagej.nih.gov/ij/download.html). Cells from triplicate wells (4 fields counted per well) were analyzed. A spreading index ((perimeter^2/4π)/actual area) was calculated

for each cell in the field and the data presented as mean spreading index ± standard deviation. Over 300 cells per data point were analyzed.

Cell migration assays. Migration assays were performed in 3 μm pore size Transwells (24 well, Costar, Cambridge, MA). Membranes in the upper chambers were pre-coated with 10 μg/ml VCAM-1 or 1 μg/ml ICAM-1 in 50 μL TBS overnight at 4°C and were then blocked with 2% BSA for 1 hour at room temperature. After washing with migration medium (RPMI-1640 supplemented with 1% FBS, 100 Units/ml penicillin and 100 μg/ml streptomycin), upper chambers were loaded with 2 x 10^5 Jurkat cells (VCAM-1) or Jurkat (α4-) cells (ICAM-1) in 160 μl of migration medium. Lower chambers contained 600 μl of migration medium supplemented with either 5 ng/ml (VCAM-1) or 1 ng/mL (ICAM-1) CXCL12 to induce chemotaxis. Cells were mixed with vehicle (1% DMSO) or 7HP349 at the indicated concentrations immediately prior to being added to the upper chamber. After a 4-hour incubation at 37°C, 5% CO_2, the upper chambers were removed, and cells in the lower chamber were collected and counted on a hemocytometer. The mean number of cells migrated in the absence of CXCL12 across VCAM-1 and ICAM-1 coated filters was 0.5 and 15 cells, respectively. These background values were subtracted from each group.

Purified α4β1 binding assays. Purified human α4β1 was immobilized (10 μg/ml) on 96-well plates in TBS (overnight at 4°C). BSA-CS-1 (400 μg/ml) was adsorbed onto fluorescent microspheres (3 μm YG beads, polysciences) also in TBS (overnight at 4°C). Both were blocked with BSA. After washing, CS-1 bound microspheres were incubated with immobilized α4β1 in the presence or absence of 7HP349 (10 μM) for 2h, RT. Unbound beads were washed away, and α4β1 bound beads were measured in a fluorescent plate reader. Function blocking mAb (HP2/1) or control IgG was used at concentrations of 10 μg/ml.

Proliferation and IL-2 production. Purified T cells (1x10^5/well) were plated in 96 well plates coated with mAb OKT3 (5 ng/well) or ICAM-1 (200 ng/well) and incubated for 2-3d in complete media at 37°C 5% CO_2 in the presence or absence of indicated concentrations of 7HP349. Proliferation was measured via standard MTT assays. Soluble IL-2 was collected and measured via ELISA assay. Function blocking antibodies or IgG control were added at the onset of culture (10 μg/ml).

Killing assay. Effector T cells (TIL) from HLA-A0201+ patients derived melanoma cell lines were incubated with HLA-matched target tumor cell line # 2767 labeled with eFluor670 at the effector-to-target ratio (E:T) of 0:1 and 1:2 and 7HP349 dose concentrations 0.01µM, 0.1µM, 1µ and 10µM for 3 hours. The cells were fixed after 3 hours incubation and permeabilized using BD Cytofix/Cytoperm, according to the manufacturer's protocol, stained with anti-cleaved caspase-3, and analyzed by LSR Fortessa X-20.

Molecular Dynamics Simulations. The α4β1 model is based on the α4β7 and α5β1 crystal structures (entries 3V4V and 3VI4, respectively) as reported previously (35). 7HP349 was initially expanded into its 3D minimized structure using BALLOON 1.6.6 and then processed with PRODRG to build topology input files. Both α4β1 and the ligand 7HP349 were prepared for docking analysis using AUTODOCK TOOLS and AUTODOCK_VINA 1.1.2. Docking was completed with exhaustiveness set to 100 and the top 20 poses evaluated. Molecular dynamics simulations were run using GROMACS with a GROMOS96 43a1 force field. The total system charge is neutralized using the appropriate number of sodium or chloride counter ions. An energy minimization is first performed to remove interatomic clashes using 500 steps of the steepest descent algorithm or until a threshold is met. Next, NVT and NPT minimization was done to prepare the complex for equilibration and production MD simulation. Trajectories for the best 3 poses were simulated for 5 nano-seconds and concatenated for analysis.

Static cell adhesion assays with CXCL12 and Pertussis Toxin. Assays testing the effect of pertussis toxin (PTX) were performed as described above using Jurkat cells and TBS with 1mM $MgCl_2$, 50% FBS. A peptide-BSA conjugate containing the connecting segment-1 (CS1) sequence of fibronectin was used as substrate (36). The concentration of CS1 used had been previously determined to be optimal for the given mediator (1 μg/mL and 5 μg/mL for 7HP349 and CXCL12, respectively). Cells were incubated with PTX at 100 ng/mL for 1.5 h prior to calcein-AM labeling. 7HP349 (30 μM) or CXCL12 (100 ng/mL) were added to the cells just prior to adding to the wells of the assay plate.

TCGA analysis. The melanoma immunotherapy cohort RNASeq data was obtained from the European Nucleotide Archive (ENA, PRJEB23709) [Pubmed: 30753825]. The raw data were quality checked with FastQC, and then mapped to human genome hg38 and transcriptome GENCODEV23 using HiSat2 [PMID: 25751142]. The gene expression level was calculated using StringTie [PMID: 25690850]. The differential expression analysis was performed between responder (CR+PR) and non-responder (PD+SD) groups using limma-trend [PMID: 24485249]. Cutoffs were set to $|logFC|>0.585$, and p-value <0.05.

Supplemental Table 1 . Immune cell frequencies were compared between the indicated treatments.

	IgG vs anti-VCAM-1	Anti-ICAM-1 vs anti-VCAM-1	IgG vs anti-VCAM-1	Anti-ICAM-1 vs anti-VCAM-1
	Vehicle		7HP349	
	**P-value**	**P-value**	**P-value**	**P-value**
**pDC**	0.22939	0.48048	0.01998	0.00613
**cDC1**	0.11219	0.08171	0.49691	0.07201
**cDC2**	0.89032	0.46829	0.00496	0.00079
**IM**	0.13143	0.51009	0.31898	0.01927
**M1Φ**	0.03353	0.91938	0.68016	0.02268

Mice bearing 3-day s.c. B16.BL6 melanomas received GVAX intradermally (i.d.) and anti-CTLA-4 (i.p.) at days 3, 6, and 9 and vehicle or 7HP349 at days 3, 4, 5, 6, and 7, and IgG, anti-VCAM-1, or anti-ICAM-1 treatment at days 3, 5, 7, 9 and 11.

[1]Immune cell frequencies were compared between dendritic cells, inflammatory monocytes and M1 macrophages after mice were treated with indicated treatments.

Supplemental Table 2.

Sources and catalog numbers for experimental animal, cell lines, flow cytometer and CyTOF antibodies, therapeutic antibodies, reagents and recombinant proteins.

REAGENT or RESOURCE	SOURCE	Cat No.
**Experimental models: Organisms/strains**		
Mouse: C57BL/6	Charles River	027
Mouse: Balb/c	Charles River	028
**Experimental models: Cell lines**		
B16.BL6	A gift from P.M. Sharma	University of Texas MD Anderson Cancer Center, Houston, TX
B16-GM-CSF	A gift from P.M. Sharma	University of Texas MD Anderson Cancer Center, Houston, TX
CT26	ATCC	Cat# CRL-2638
E.G7-OVA	ATCC	Cat# CRL-2113
LLC	A gift from Dr. M.A. Cortez	University of Texas MD Anderson Cancer Center, Houston, TX
Jurkat cell line	ATCC	TIB-152
Jurkat cell line (K562)	ATCC	CCL-243
Jurkat cell line (HSB-2)	ATCC	CLL-120.1
Jurkat cell lines (RBL-1)	ATCC	CRL-1378
Jurkat cell lines (70Z/3)	ATCC	TIB-158
Jurkat cell lines (DH82)	ATCC	CRL-10389
**Antibodies**		
Anti-mouse Ly-6C (clone HK1.4)	BioLegend	128002
Anti-mouse CD4 (clone RM4-5)	BioLegend	100506
Anti-mouse CD11b (clone M1/70)	BioLegend	101249
Anti-mouse Ly-6G (clone 1A8)	DVS-Fluidigm	3141008B
Anti-mouse CD11c (clone N418)	DVS-Fluidigm	3142003B
Anti-mouse CD183 (clone CXCR3-173)	BioLegend	126502
Anti-mouse IL-2 (clone JES6-5H4)	DVS-Fluidigm	3144002B
Anti-mouse CD69 (clone H1.2F3)	DVS-Fluidigm	3145005B
Anti-mouse CD8a (clone 53-6.7)	BioLegend	100702
Anti-mouse CD223, LAG-3 (C9B7W)	BioLegend	125202
Anti-mouse CD19 (clone 4D5)	BioLegend	115502
Anti-mouse CD25 (clone 3C7)	DVS-Fluidigm	3150002B
Anti-mouse p-Stat3 (Tyr705) (clone M9C6)	Cell Signaling Tech.	4113S
Anti-mouse CD49d (clone MFR4.B)	BioLegend	103701
Anti-mouse CD274, PD-L1 (clone 10F.9G2)	BioLegend	124337
Anti-mouse CD272, BTLA (clone 6F7)	DVS-Fluidigm	3156028B
Anti-mouse Foxp3 (clone FJK-16s)	DVS-Fluidigm	3158003A
Anti-mouse F4/80 (clone BM8)	DVS-Fluidigm	3159009B

Anti-mouse CD44 (clone IM7)	BioLegend	103002
Anti-mouse T-bet (clone 4B10)	DVS-Fluidigm	3161014B
Anti-mouse Bax (clone 5B7)	BioLegend	633702
Anti-mouse CD62L (clone MEL-14)	DVS-Fluidigm	3164003B
Anti-mouse IFNg (clone XMG1.2)	DVS-Fluidigm	3165003B
Anti-mouse Bcl-2 (clone BCL/10C4)	BioLegend	633502
Anti-mouse IL-6 (clone MP5-20F3)	DVS-Fluidigm	3167003B
Anti-mouse RORgt (clone 600214)	DVS-Fluidigm	3168018B
Anti-mouse p-Jak2 (clone C80C3)	Cell Signaling Tech.	3776
Anti-mouse CD49b, Integrin α2 (clone HMα2)	DVS-Fluidigm	3170008B
Anti-mouse CD279, PD-1 (clone 29F.1A12)	BioLegend	135202
Anti-mouse Ki67 (clone B56)	DVS-Fluidigm	3172024B
Anti-mouse Granzyme B (clone GB11)	DVS-Fluidigm	3173006B
Anti-mouse IL-17A (clone TC11-18H10.1)	BioLegend	506935
Anti-mouse CD127, IL-7Ra (clone A7R34)	DVS-Fluidigm	135029
Anti-mouse CD278, ICOS (clone 7E.17G9)	eBioscience	14-9942-85
Anti-mouse I-A/I-E, MHC-II (clone M5/114.15.2)	DVS-Fluidigm	3209006B
Anti-mouse CD278, ICOS (clone C398.4A)	Biolegend	313502
Anti-mouse CD357, GITR (clone DTA1)	DVS-Fluidigm	3143019B
Anti-mouse GATA3 (clone TWAJ)	eBioscience	14-9966-82
Anti-mouse Ly-6G (clone 1A8)	Biolegend	127637
Anti-mouse CXCR2 (clone SA044G4)	Biolegend	149302
Anti-mouse CD197, CCR7 (clone SA044G4)	eBioscience	16-1971-85
Anti-mouse CD69 (clone H1.2F3)	Biolegend	104533
Anti-mouse CD357, GITR (clone CXNFT)	DVS-Fluidigm	3161011B
Anti-mouse TNFa (clone MP6-XT22)	DVS-Fluidigm	3162002B
Anti-mouse Ki67 (clone B56)	BD Biosciences	556003
Anti-mouse CD206, MMR (clone C068C2)	DVS-Fluidigm	3169021B
Anti-mouse CD127, IL-7Ra (clone A7R34)	Biolegend	135029
Anti-mouse B220, CD45R (clone RA3-6B2)	Biolegend	103202
Rat IgG1 isotype (clone HPRN)	BioXcell	BE0088
Anti-mouse CD11a-PE (clone M17/4)	eBioscience	12-0111-82
Anti-mouse TNFa□BV650□□clone MP6-XT22)	BD Biosciences	563943
Anti-mouse CD11c-PE-Cy7 (clone N418)	Biolegend	117318
Anti-mouse CD11c-BV421 (clone N418)	Biolegend	117329
Anti-mouse CD11b-AF700 (clone M1/70)	eBiosciences	56-0112-82
Anti-mouse Ly6G-BUV395 (clone 1A8)	BD Biosciences	563978
Anti-mouse CD19-PE Cy5 (clone MB19-1)	Biolegend	115510
Anti-mouse granzyme B-FITC (clone NGZB)	eBiosciences	11-8898-809
Anti-mouse granzyme B-Efluor 660 (clone NGZB)	eBioscience	50-8898-82
Anti-mouse CCR7-PE Cy7 (clone 4B12)	Biolegend	120124
Anti-mouse CCR6-BV605 (clone 29-2L17)	Biolegend	129819
Anti-mouse Foxp3-APC (clone FJK-16s)	Invitrogen	17-5773-82
Anti-mouse Ki-67-PE Cy7 (clone 16A8)	Biolegend	652426
Anti-mouse Ki-67-PE (clone 16A8)	Biolegend	652403
Anti-mouse CD45-BV421 (clone 30-F11)	Biolegend	103133
Anti-mouse CD45-APC Cy7 (clone 30-F11)	Biolegend	103116
Anti-mouse CD45-AF700 (clone 30-F11)	Biolegend	103128

Anti-mouse CD44-FITC (clone IM7)	Biolegend	103022
Anti-mouse CD44-PE-Cy7 (clone IM7)	Biolegend	103029
Anti-mouse CD4-BV785 (clone GK1.5)	Biolegend	100453
Anti-mouse F4/80-APC (clone BM8)	Biolegend	123116
Anti-mouse F4/80-BV421 (clone BM8)	Biolegend	123132
Anti-mouse PD-L1-PE Cy7 (clone 10F.9G2)	Biolegend	124314
Anti-mouse Bcl-2-Alexa Fluor 647 (clone BCL/10C4)	Biolegend	633510
Anti-mouse Lag3-BV650 (clone C9B7W)	Biolegend	125227
Anti-mouse PD-1-APC Cy7 (clone 29F.1A12)	Biolegend	135224
Anti-mouse Tim-3-PE (clone B8.2C12)	Biolegend	134003
Anti-mouse ICOS-FITC (clone C398.4A)	Biolegend	313506
Anti-mouse CXCR3-APC (clone CXCR3-173)	Biolegend	126511
Anti-mouse Ly6c-BV605 (clone AL-21)	BD Biosciences	563011
Anti-mouse Ly6c-APC (clone AL-21)	BD Biosciences	560595
Anti-mouse CD25-FITC (clone 3C7)	Biolegend	102005
Anti-mouse Pstat3-Percp-Cy5.5 (clone 4/P-Stat3)	BD Biosciences	560114
Anti-mouse IL-17A-APC Cy7 (clone TC11-18H10.1)	Biolegend	506940
Anti-mouse T-bet-BV421 (clone 4B10)	Biolegend	644815
Anti-mouse T-bet-PE (clone 4B10)	BD Biosciences	561265
Anti-mouse CD206-AF647 (clone C068C2)	Biolegend	141712
Anti-mouse CD103-PE (clone 2E7)	eBioscience	12-1031-81
Anti-mouse CCR2-BV510 (clone SA203G11)	Biolegend	150617
Anti-mouse CXCL9-AF 647 (clone MIG-2F5.5)	Biolegend	515606
Anti-mouse CCL5-PE (clone 2E9/CCL5)	Biolegend	149104
Anti-mouse CCL20-AF 700 (clone 114906)	R&D	IC760N
Anti-mouse RORgt-BV650 (clone Q31-378)	Fisher Scientific	BDB564722
Anti-mouse CD8 - BV711 (clone 53-6.7)	Biolegend	100747
Anti-mouse CTLA-4 mouse IgG (clone 9H10)	Bioxcell	BP0131
Anti-mouse PD-1 mouse IgG2A,K (clone RMP1-14)	Bioxcell	BP0146
Anti-mouse CD8 (clone clone 53-6.7)	Bioxcell	BE0004-1
Anti-mouse NK1.1 (clone PK136)	Bioxcell	BP0036
Anti-mouse ICAM-1 (clone YN1/1.7.4)	Bioxcell	BE0020-1
Anti-mouse VCAM-1 (clone M/K-2.7)	Bioxcell	BE0027
Anti-mouse Ly6G (clone 1A8)	Bioxcell	BE0075-1
Anti-mouse CXCL12 (clone 79014)	Bioxcell	MAB310-500
Mouse IgG isotype (clone N/A)	Bioxcell	BE0087
Rat IgG2a,□ isotype (clone RMP1-14)	Bioxcell	BP0146
Rat IgG1 isotype (clone HPRN)	Bioxcell	BE0088
Anti-human a4 (clone HP2/1)	Bio-Rad	MCA697
Anti-human aL (clone 38)	Bio-Rad	MCA1848
Anti-human b1 (clone P5D2)	R&D Systems	MAB17781
Anti-human ICAM-1 (clone BBIG-I1)	R&D Systems	BBA3
Anti-human a5 (clone SAM-1)	Thermo Fisher Scientific	14-0496-82
Anti-human MAdCAM-1 (clone MECA-367)	Thermo Fisher Scientific	16-5997-85
Anti-human b7 (clone FIB27)	BD Biosciences	553812,
Anti-human b1 (clone HUTS-21)	BD Biosciences	556047
Anti-human b1 (clone 9EG7)	BD Biosciences	553715

Anti-human a4 (clone L25)	BD Biosciences	747299,
Anti-human b1 (clone B44)	Millipore	MAB2259Z,
Anti-human b2 (clone MEM-48)	AbCAM	AB657,
Donkey anti-goat polyclonal	Thermo Fischer Scientific	A-21447
Anti-human b1 (clone 33B6)	A gift from Dr. Bradley McIntyre	University of Texas MD Anderson Cancer Center, Houston, TX
**Chemicals, peptides, and recombinant proteins**		
Human collagen type I	Sigma-Aldrich	CC050
Human collagen type IV	Sigma-Aldrich	CC076
Fibronectin	Sigma-Aldrich	F2006
Fibrinogen	Sigma-Aldrich	341576
Human VCAM-1	R&D Systems	ADP5-200
Human ICAM-1	R&D Systems	ADP4-200
Human MAdCAM-1	R&D Systems	6056-MC-050
Human CXCL12	R&D Systems	350-NS-050
Mouse VCAM-1	R&D Systems	643-VM-200
Cell-ID Intercalator-Ir	Fludigm	201192A
Cell-ID^{TM} Cisplatin	Fludigm	201064
RPMI 1640	Corning	10-040-CV
Penicillin Streptomycin	Corning	30-002-CI
0.05% Trypsin, 0.53nM EDTA	Thermo Fisher	25-052-CI
Histopaque	Sigma-Aldrich	11191
Phosphare Buffered Saline	Corning	21-040-CV
ACK Lysing Buffer	Gibco	A10492-01
Fetal Bovine Serum	Gemni Bio-Products	110-106
Tyramide Signal Amplification	Fortis Life Science	www.fortislife.com
Foxp3/ Transcription factor staining buffer set	eBioscience	00-5523-00
AllPrep® DNA/RNA Kit	Qiagen	80284
Mouse SDF-1 alpha / CXCL12 alpha ELISA kit	Sigma-Aldritch	RAB0125-1KT
MACS Pan T cell Isolation Kit	Miltenyi Biotec	130-096-535
Calcein-AM	Thermo Fisher	C1430
Maxpar Fix and perm buffer	Fludigm	201067
H-2Kb – KSPWFTTL peptide	Peptide Internatiional	https://www.bio-connect.nl/supplier/vivitide/
H-2Kb – SVYDFFVWL peptide	Peptide Internatiional	https://www.bio-connect.nl/supplier/vivitide/
H-2D^{b} – KVPRNQDWL peptide	CPC scientific	https://cpcscientific.com/
